# Serotonergic Mechanisms in Proteinoid-Based Protocells

**DOI:** 10.1021/acschemneuro.4c00801

**Published:** 2025-01-22

**Authors:** Panagiotis Mougkogiannis, Andrew Adamatzky

**Affiliations:** Unconventional Computing Laboratory, University of the West of England, Bristol BS16 1QY, U.K.

**Keywords:** consciousness, proteinoids, protocells, serotonin, paroxetine, neurotransmitters, origin of life, artificial cells, primordial soup, protocellular consciousness

## Abstract

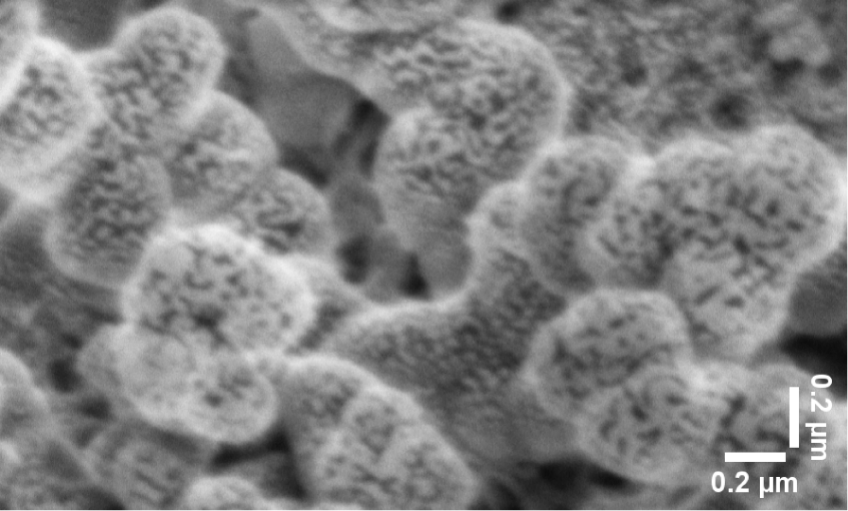

This study examines the effects of incorporating serotonin (5-HT) into proteinoid microspheres. It looks at the microspheres’ structure and electrochemical properties. Proteinoid-serotonin assemblies have better symmetry and membrane organization than pristine proteinoids. Cyclic voltammetry shows a big boost in electron transfer. This is proven by a smaller peak separation and higher electrochemical efficiency. SEM imaging shows a distinct core–shell structure and uniform density. This suggests ordered molecular assembly. These findings show that serotonin changes proteinoid self-assembly. It creates structured systems with better electron transfer pathways. The serotonin-modified proto-neurons show new properties. They give insights into early cellular organization and signaling. This helps us understand prebiotic information processing systems.

## Introduction

The rise of cellular life required self-organizing molecular systems. They had to maintain chemical gradients and process information.^1^ Proteinoids are a model for studying prebiotic cellular evolution. They form by thermally condensing amino acids. They spontaneously create membrane-like microspheres and have biomimetic properties.^2^ These structures closely resemble modern cells. They can sequester molecules and maintain chemical differences across their boundaries.^3^

Fox theorized that proteinoids are primitive proto-neurons. They formed from amino acids through thermal copolymerization. They could have been key to the origin of life.^4,5^ When immersed in water, these thermal proteins self-organize into microspheres. They have membrane-like boundaries and basic, cell-like properties.^6,7^ These traits suggest proteinoids as possible precursors to modern cells. They could compartmentalize and perform primitive metabolic activities.^8^

We use proteinoids’ unique self-assembling, functional properties in our study. We incorporate serotonin, a key neurotransmitter.^9^ The rationale for combining proteinoids with serotonin is their ability to form stable microspheres. They could serve as drug delivery vehicles.^10,11^ Their amino acid composition gives them high biocompatibility. They can also encapsulate and protect bioactive molecules. So, they are ideal for delivering neurologically active compounds like serotonin.^12,12^ This approach builds on Fox’s work on proteinoids’ self-assembly.

**Figure 1:**
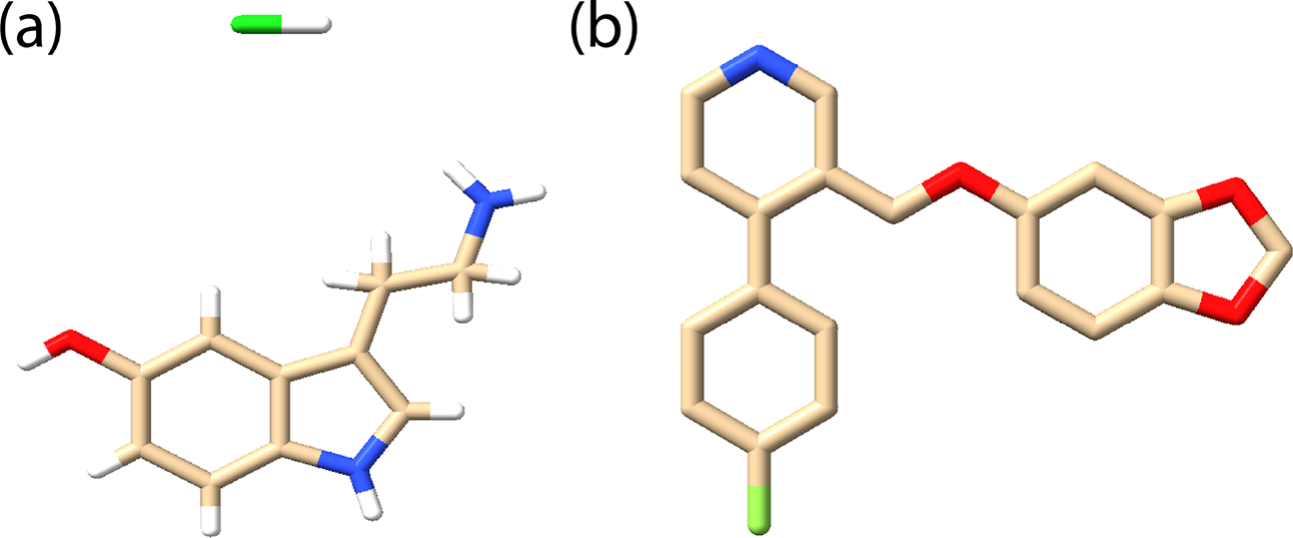
Molecular structures of (a) serotonin hydrochloride (5-hydroxytryptamine hydrochloride, C_10_H_12_N_2_O·HC_*l*_) and (b) paroxetine ((3*S*,4*R*)-3-[(2*H*–1,3-benzodioxol-5-yloxy)methyl]-4-(4-fluorophenyl)piperidine, C_19_H_20_FNO_3_). Color coding: carbon (beige), hydrogen (white), nitrogen (blue), oxygen (red), fluorine (green), and chloride counterion (green). The chemical visualization of serotonin hydrochloride and paroxetine was conducted using ChimeraX.^39^

Using bioactive molecules in proteinoid systems may help us understand primitive cellular functions. Serotonin (5-hydroxytryptamine, 5-HT) (Figure 1a) is a key neurotransmitter. Its unique chemicals make it important for proto-cellular studies. Its aromatic structure and ability to participate in π–π stacking interactions contribute to molecular organization patterns similar to those observed in contemporary membrane proteins.^13^ Serotonin’s amphipathic nature lets it affect membrane organization in cells. This suggests it may help in primitive cellular assembly.^14^

Recent studies on prebiotic molecular self-assembly show that aromatic amino acids are key to forming ordered structures.^15^ Indole rings, found in tryptophan and serotonin, promote stable molecular structures. They do this through noncovalent interactions. Studies of modern neurotransmitter vesicles show that aromatic parts help. They organize and stabilize membranes.^16^ This gives insights into potential mechanisms in proteinoid systems.

The electrochemical properties of serotonin offer additional perspectives on proto-cellular function. In modern biology, serotonin helps with electron transfer and redox balance.^17^ These traits suggest a role in primitive energy processes. This is true, especially for proteinoid structures that can maintain chemical gradients. Measuring these electrochemical processes gives insights into proto-cellular systems. It provides a way to quantify their functionality.^18^

Studying serotonergic mechanisms in proteinoid systems may fill gaps in our knowledge of cellular evolution. Modern cells use complex proteins to store and release neurotransmitters. But, simpler systems might have preceded these mechanisms.^19^ Studying serotonin-proteinoid interactions may reveal the origins of cellular organization and the evolution of neurotransmitter systems. It could shed light on pathways from prebiotic chemistry to modern biology.

Serotonin affects human mood through a complex network of biological pathways. Serotonin affects neuronal plasticity in the prefrontal cortex (PFC). It does this by activating 5-HT_2_ receptors. This increases brain-derived neurotrophic factor (BDNF).^20^ This process is similar to other monoamine neurotransmitters, like dopamine and norepinephrine. Aromatic rings enable distinct molecular recognition patterns.^21^ The serotonergic system has unique patterns in mood modulation over time and space. Neurons in the raphe nuclei secrete serotonin. This uses a volume transmission mechanism. It influences many brain regions through extrasynaptic diffusion.^22^ This diffuse signaling strategy is like that of other amphipathic macromolecules, such as endocannabinoids. They also influence brain networks by volume transfer.^23^ Serotonin can change membrane properties over large areas. This mirrors its effects in proteinoid systems. It suggests important physicochemical features that go beyond specific biological settings. Also, serotonin regulates circadian rhythms. This offers insights into basic cellular timing systems. Serotonin controls circadian gene expression by interacting with 5-HT_7_ receptors in the suprachiasmatic nucleus (SCN).^24^ This timing system is like bacterial quorum sensing. There, aromatic compounds, like homoserine lactones, control group behaviors.^25^ The timing of serotonin signaling may reflect advanced molecular clocks. Depression and anxiety disorders involve complex links between serotonin and the hypothalamic-pituitary-adrenal (HPA) axis, a major neuroendocrine system regulating stress response and mood regulation. Chronic stress alters serotonin receptor expression and trafficking. It does so via pathways that involve reorganizing membrane lipids.^26^ Other stress-related chemicals, like glucocorticoids, have similar effects on membranes. This suggests shared principles in membrane-mediated cellular responses.^27^ Understanding these membrane-level interactions sheds light on brain function and potential stress responses. The interplay between serotonin and the immune system reveals more biological complexity. Serotonin regulates inflammation. It does this by affecting immune cells and cytokines.^28^ This neuroimmune interface resembles ancient chemical signaling systems in primitive species, like slime molds. In those, cyclic adenosine 3′,5′-monophosphate (cAMP) is both a messenger and a communication molecule.^29^ Serotonin’s dual role in the brain and immune system may hint at ancient links between chemical signaling systems (Table 1).

**Table 1:** Comparative Analysis of Molecular Signaling Mechanisms across Biological Systems

Signaling Feature	Modern System	Primitive Analog
Volume transmission	Serotonin diffusion in brain^30^	Quorum sensing molecules
Temporal organization	Circadian rhythm regulation^31^	Bacterial timing systems
Stress response	HPA axis modulation^32^	Protocellular adaptation
Immune integration	Neuroimmune signaling^33^	Primitive chemical messaging

**Figure 2:**
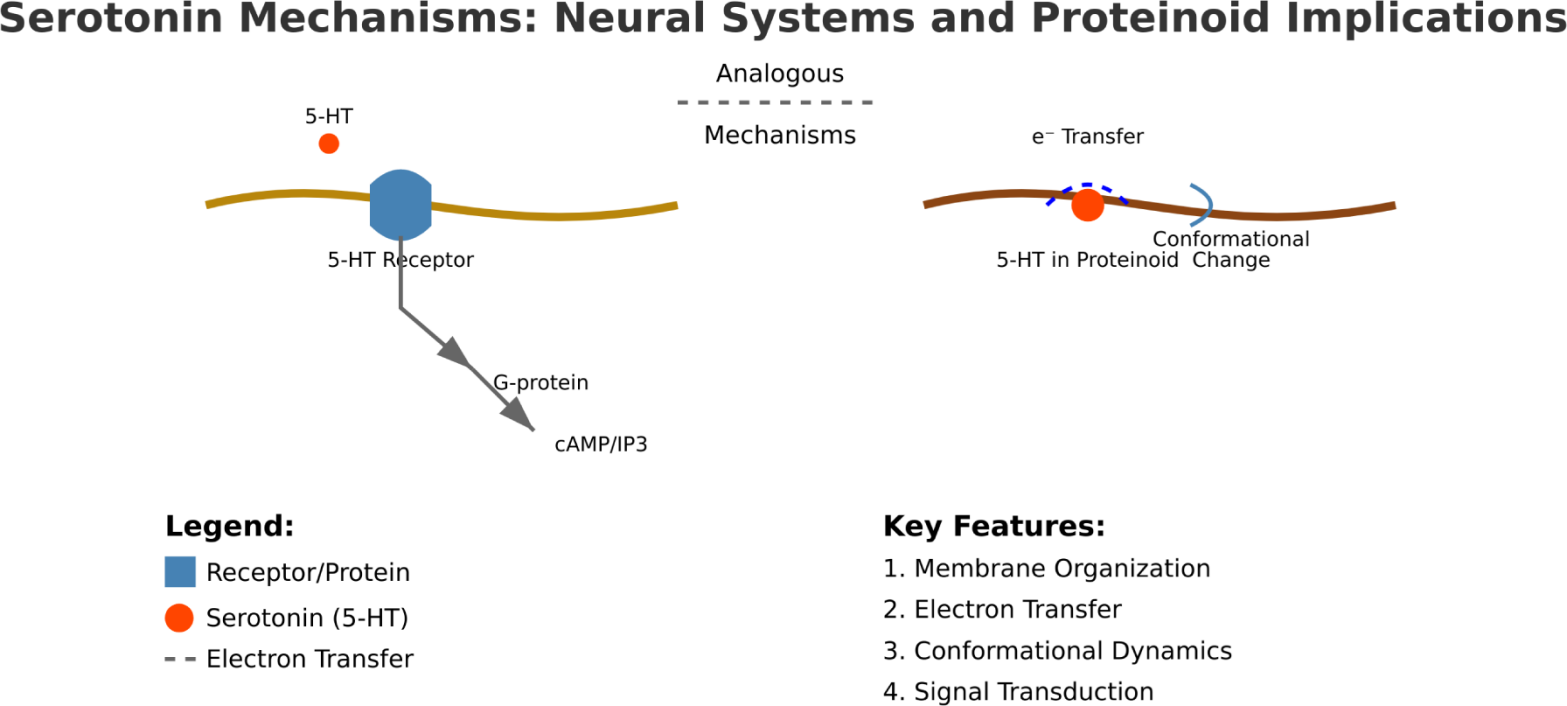
Integration of serotonin mechanisms in neural systems and proteinoid structures. The schematic illustrates parallel mechanisms between modern neural signaling and primitive proteinoid systems. In neural systems (left panel), serotonin (5-HT) binds to membrane receptors (5 -HT_1—7_). This starts G-protein signaling channels that regulate neural function via second messengers (cAMP/IP3).^40^ The membrane processes include receptor-mediated signaling, ion flux regulation, and changes in transport proteins.^41^ In proteinoid systems (right panel), serotonin molecules incorporate into the membrane-like structures. This gives them new properties. The indole ring of serotonin helps electron transfer. It does so via π–π stacking interactions, like in modern biological systems’ electron transport chains.^42^ These interactions change the proteinoid’s shape. They may be primitive versions of modern signal transduction mechanisms.^29^ The comparison reveals fundamental similarities in molecular organization and function. Key features include: (1) membrane organization via aromatic interactions, (2) enhanced electron transfer due to serotonin’s structure, (3) dynamics affected by molecular recognition, and (4) primitive signal transduction. The enhanced electron transfer efficiency in proteinoid-serotonin systems (ϵ_prot-sero_/ϵ_prot_ = 23.03) suggests that neurotransmitter-like molecules may have played crucial roles in early cellular evolution.^43^ These parallel mechanisms offer insights into two things. They are the evolution of neural signaling and the role of aromatic molecules in protocellular systems.^28^

To understand the shift from early chemistry to today’s cell functions, we must explore key molecular pathways. Figure 2 shows serotonin’s chemical properties. They can affect membrane organization and electron transfer in modern neurons and simple proteinoid structures. These mechanisms suggest possible evolutionary paths for cellular signaling systems. The selective serotonin reuptake inhibitor (SSRI) paroxetine affects serotonergic neurotransmission. It binds with high affinity (*K_i_* = 0.13 nM) to the serotonin transporter (SERT).^34^ The treatment for major depression works by blocking serotonin reuptake. This raises serotonin levels in the brain. Crystal structures of SERT-paroxetine complexes (Figure 1b) have shown unique interactions in the binding pocket. Notably, they involve Asp98 and Tyr95. These interactions enhance paroxetine’s binding affinity and selectivity.^35^ Paroxetine binding changes SERT’s shape. It stabilizes an outward-open state. This inhibits serotonin reuptake.^36^ Long-term use of paroxetine changes serotonin receptor levels and sensitivity. It notably affects 5-HT1*A* autoreceptors.^37^ The molecular processes explain the delayed therapeutic effects seen in clinics. They indicate complex changes in serotonergic neurotransmission.^38^

This study explores serotonergic mechanisms in proteinoid-based protocells. It focuses on how serotonin and paroxetine affect their structure and function. We present a comprehensive analysis using many methods. First, we use a scanning electron microscope to examine the architecture of proteinoid complexes. We do this before and after adding serotonin/paroxetine. This reveals distinct microscale structures. Second, we use cyclic voltammetry to measure redox behavior in different proteinoid modifications. Third, we analyze spiking patterns in voltage measurements. This shows how neuroactive compounds affect the protocells’ electrical responses. Additionally, we use FTIR spectroscopy to confirm successful molecular incorporation and structural changes. We aim to use a multimodal characterization approach. It will give insights into using neurotransmitter-based mechanisms in synthetic protocellular systems. This may advance our understanding of minimal, neural-like networks and bioinspired computing.

## Methods and Materials

The synthesis of proteinoids used various analytical grade amino acids. These were l-glutamic acid (l-Glu, CAS-No: 56-86-0), l-phenylalanine (l-Phe, CAS-No: 63-91-2). All were purchased from Sigma-Aldrich Ltd., UK, with reagent grade >98%. Serotonin hydrochloride (H9523, ≥98% purity, powder form) and paroxetine hydrochloride hemihydrate (C_19_H_20_FNO_3_·HCl·0.5H_2_O, MW: 374.83, CAS: 110429-35-1) were purchased from Merck-Sigma-Aldrich. The drug paroxetine, also known as (3*S*-*trans*)-3-[(1,3-benzodioxol-5-yloxy)methyl]-4-(4-fluorophenyl)piperidine hydrochloride hemihydrate, was used as received.

The proteinoid solutions were prepared using both water and NaCl 0.15 M ionic solution. Proteinoids (l-Glu:l-Phe) were made by heating equal amounts of l-glutamic acid and l-phenylalanine, each at 2.5 g. The amino acid mixture was heated to its melting point in a reflux apparatus under continuous stirring. This will yield a homogeneous slurry. The molten mass was cooled to 80 °C and diluted with deionized water, followed by stirring for 3 h. The precipitate was obtained through vacuum filtration. The purified proteinoid powder was obtained via lyophilization. Characterization of the proteinoid structures was performed using Fourier Transform Infrared (FT-IR) spectroscopy on a Nicolet iS 5 FTIR Spectrometer (Thermo Scientific). The spectra were collected across a scan range of 400 to 4000 cm^–1^ with a resolution of 4 cm^–1^. The FTIR measurements were conducted by depositing different proteinoid solutions on the spectrometer crystal.

Data collection and spectrum analysis were carried out using the Bicolet Omnic program (OMNIC Series Software, Thermo Scientific). The FT-IR analysis revealed peaks at 1635, 1943, 2108, 2349, and 3258 cm^–1^ (more information in Figure S1 and Table S1). Specific peaks matched the amide I and II bands, which are characteristic of the peptide backbone. The amide II band at 1635 cm^–1^ is from vibrations of peptide bonds between amino acids. The peak at 1943 cm^–1^ corresponds to the amide I band, which is from stretching of the peptide group bonds.^44^

**Figure 3:**
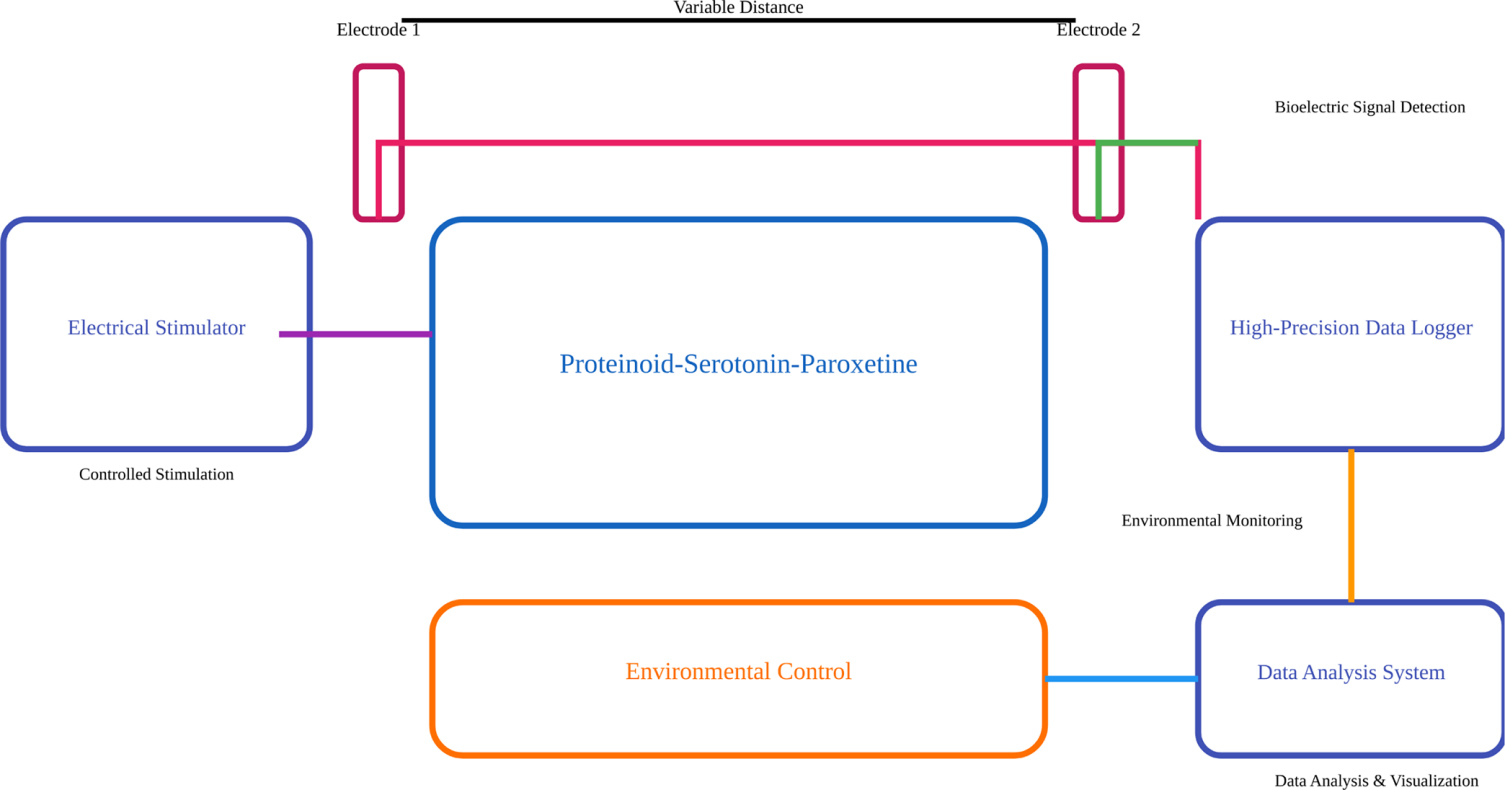
Schematic representation of the proteinoid-serotonin-paroxetine bioelectric measurement system. The setup employs dual iridium–platinum electrodes (diameter: 0.1 mm) positioned 10 mm apart for potential difference measurements. Bioelectric signals are captured using an ADC-24 PicoLog high-precision data logger. The system integrates controlled electrical stimulation, environmental monitoring, and real-time data analysis. The environmental control unit ensures stable measurement conditions. The data analysis system enables signal processing and visualization. The electrodes detect bioelectric signals in the proteinoid-serotonin-paroxetine complex. They do this while meeting controlled stimulation parameters.

Figure 3 shows the setup for measuring bioelectric signals from the proteinoid-serotonin-paroxetine system. The apparatus had two iridium–platinum electrodes, 0.1 mm in diameter. They were fixed 10 mm apart. Bioelectric potential differences were measured using an ADC-24 PicoLog system. It has high-precision voltage measurement capabilities. The setup included an electrical stimulator to precisely excite the sample. An environmental control system ensured stable conditions during the tests. The data acquisition system was set up for real-time monitoring and subsequent analysis of bioelectric signals. This setup allowed for accurate detection of changes in the proteinoid-serotonin-paroxetine complex under controlled stimulation.

Electrical characterization measurements were conducted utilizing an Ossila potentiostat with high-precision specifications. The instrument offered a potential range of ±7.5 V, with a compliance voltage of ±10 V and an applied potential accuracy of ±10 mV offset. The potentiostat controlled current measurements with high precision. It had five settings, with ranges from ±20 nA to ±200 mA. It achieved a 5 nA resolution at the 20 μA range. The instrument’s high resolution and accuracy (±20 nA at 20 μA) enabled precise detection of subtle changes in the electrical properties of the proteinoid-serotonin-paroxetine complexes. We achieved data acquisition via USB-B communication. It ensured reliable, continuous monitoring during the experiments.

The electrochemical impedance spectroscopy (EIS) measurements were performed using a Zimmer Peacock potentiostat. The measurements were done at a DC potential (Edc) of 0.1 V and an AC amplitude (Eac) of 0.01 V. The frequency range was swept from 0.00001 Hz to 1,000,000 Hz at 12.3 points per decade, for a total of 136 points. An equilibration time of 10 s was maintained. Measurements were taken using a fixed frequency scan versus open circuit potential (OCP).

We used high-resolution SEM on an FEI Quanta 650 to characterize the proteinoids. Before imaging, we sputter-coated the samples with a thin layer of gold. This protected the specimens and provided the necessary conductivity for imaging. This coating process made a stable charged-particle beam. It is key for high-resolution surface topography. The FEI Quanta 650s advanced imaging allowed a detailed, nanometer-scale view of the proteinoid structures. It revealed their surface features, internal architecture, and dimensions.

## Results

### Morphological Characterization of Proteinoid-Serotonin-Paroxetine

**Figure 4:**
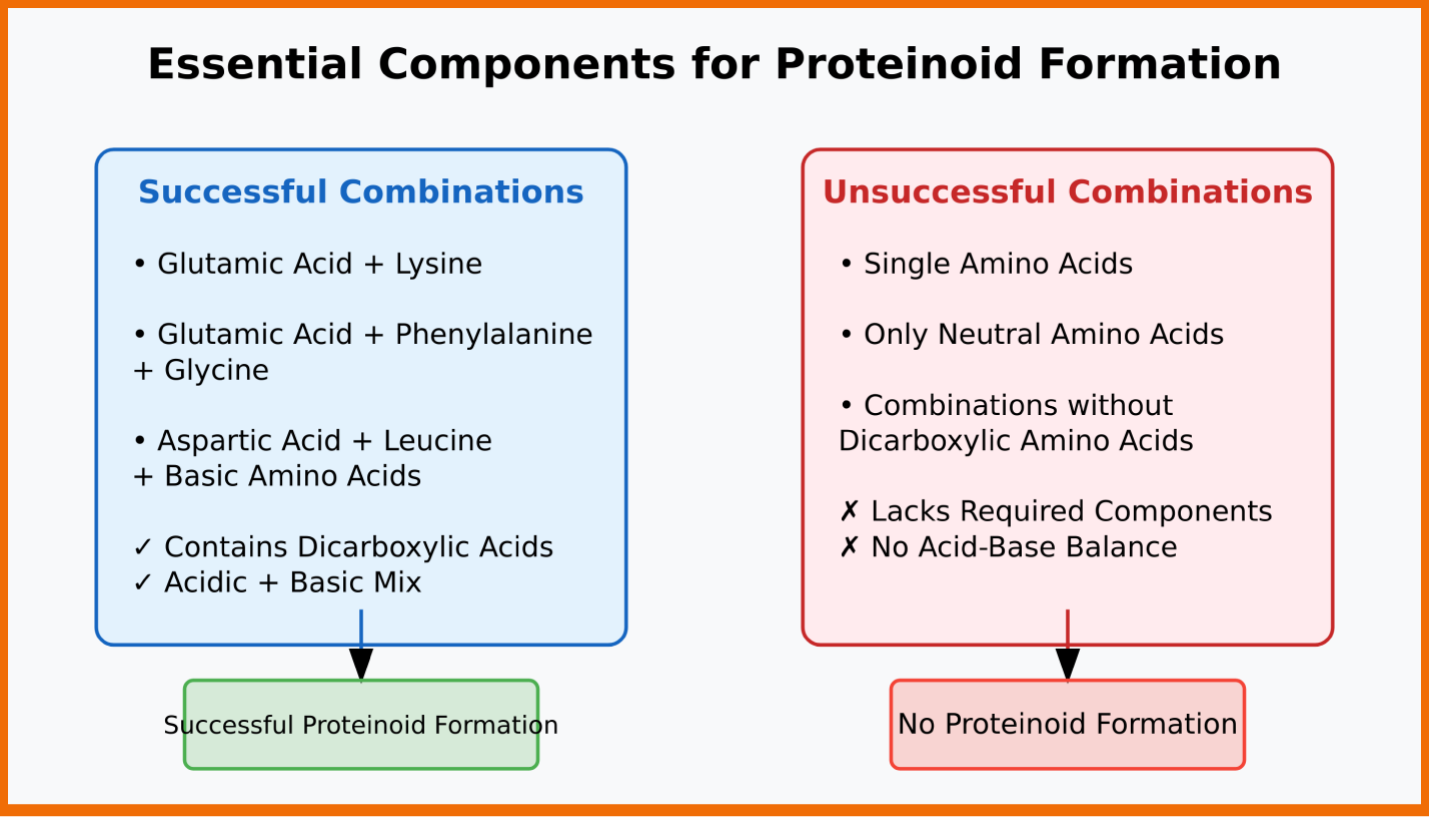
Schematic representation of amino acid mixtures determining successful proteinoid formation. The left panel (blue) shows effective combinations. They require dicarboxylic amino acids (e.g., glutamic acid) paired with basic amino acids (e.g., lysine) or three-component mixtures. The right panel (red) shows failed combinations. They lack dicarboxylic acids or have an improper acid–base balance. Successful combinations create thermal proteinoids (green). Ineffective ones do not (red).

Proteinoids can form only with specific amino acid combinations (Figure 4). Dicarboxylic amino acids are crucial for this. Successful combinations include glutamic acid with lysine. They show the need to mix acidic and basic amino acids. Also, three-component mixtures, like glutamic acid-phenylalanine-glycine or aspartic acid-leucine-basic amino acids, yield effective proteinoid formation. Combinations that lack dicarboxylic amino acids, or contain only neutral amino acids, or consist of single amino acids alone, fail to form proper proteinoid structures.^45^

Serotonin’s function in the brain extends beyond mere neurotransmission. It regulates membrane fluidity and cellular signaling with great precision. The compound has significant flexibility throughout biological systems. It engages with many receptor types (5-HT1–7) and voltage-gated ion channels.^46^ In proteinoid systems, serotonin appears to exert analogous effects on membranes. Our investigation revealed this through enhanced electrochemical responses and fluctuating potential variations. The comparison of serotonin signaling and proteinoid-serotonin behavior suggests that some key traits transcend different molecules. The molecular architecture of serotonin transporter proteins (SERT) exhibits specific binding sites. Aromatic interactions are crucial for substrate recognition and transport mechanisms.^47^

**Figure 5:**
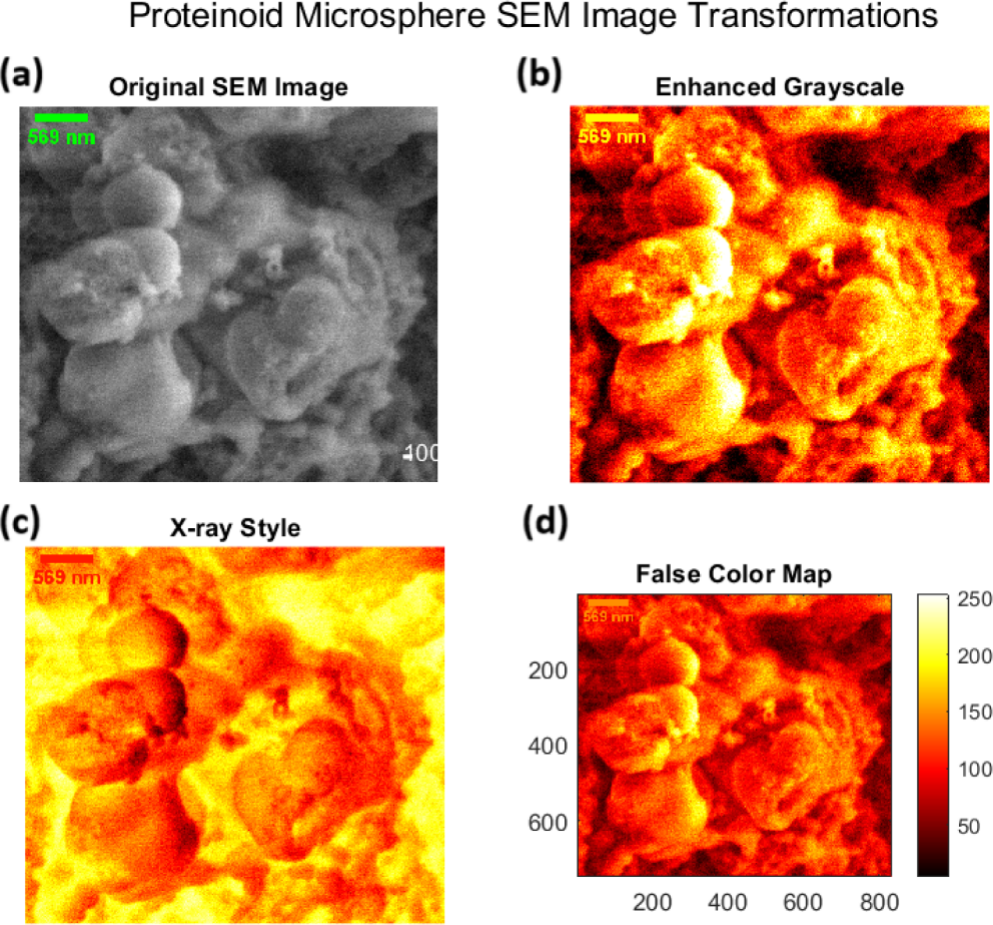
SEM image transformations of proteinoid microspheres revealing structural characteristics at multiple contrast levels. (a) Original SEM micrograph showing spherical proteinoid assemblies with diameter 589 ± 42 nm and surface roughness features. (b) Enhanced grayscale transformation highlighting topographical variations and interface boundaries between microspheres, revealing subtle surface texturing. (c) X-ray style visualization emphasizing density gradients and internal structural features through inverted contrast, particularly evident at microsphere interfaces. (d) False color mapping with intensity scale (0–250 arbitrary units) providing quantitative visualization of height variations and surface morphology. Scale bar: 400 nm. The complementary image transformations reveal hierarchical organization from nano- to microscale, with distinct boundary regions (∼50–100 nm) between adjacent microspheres and surface roughness features (∼20–50 nm) distributed across individual spheres.

**Figure 6:**
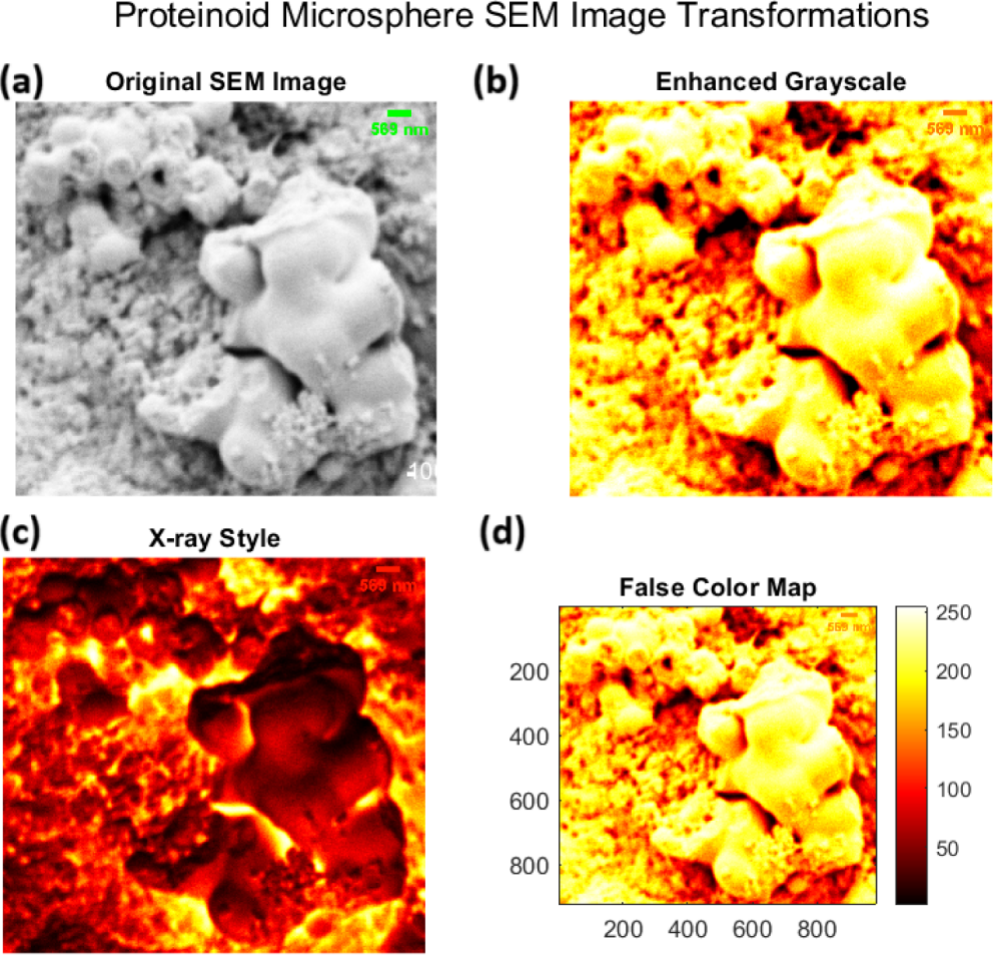
Multimodal visualization of proteinoid microsphere ultrastructure through SEM image transformations. (a) Original SEM micrograph revealing microsphere assemblies (length scale: 589 nm) with characteristic surface topology and intersphere fusion zones. (b) Enhanced grayscale rendering highlighting morphological gradients and surface roughness patterns, with bright regions (>200 intensity units) corresponding to elevated features. (c) X-ray style inversion emphasizing internal density distributions and boundary interfaces (∼50–100 nm thickness), with dark regions indicating higher electron density. (d) Quantitative false color mapping (0–250 arbitrary units) with spatial calibration (800 × 800 pixels) revealing hierarchical organization: primary spheres (∼500–600 nm), interconnecting regions (∼100–200 nm), and nanoscale surface features (∼20–50 nm). The sequential transformations reveal a complex topographical landscape with distinct structural hierarchy spanning 3 orders of magnitude. Scale bar: 50 nm.

**Figure 7:**
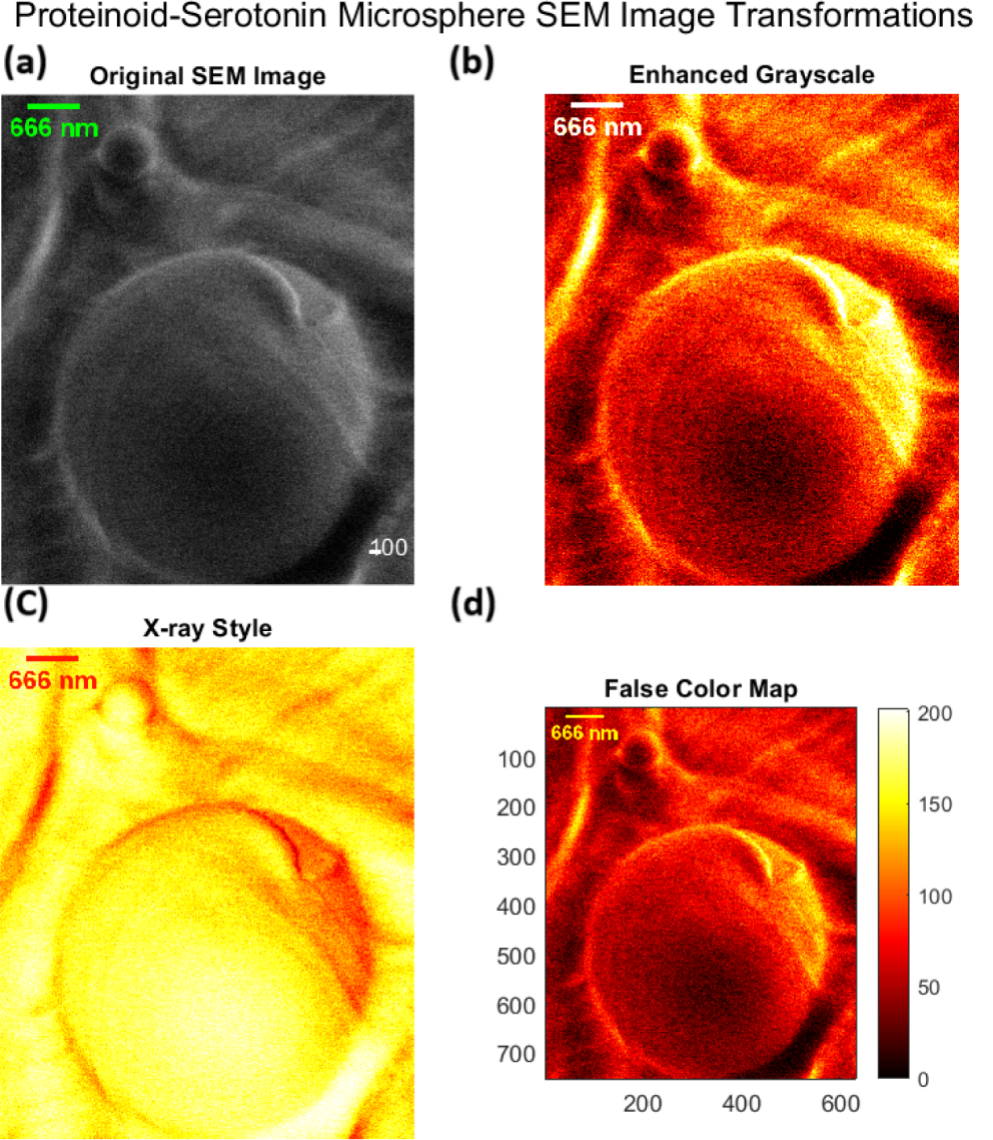
Advanced SEM visualization of proteinoid-serotonin-paroxetine microsphere revealing distinctive morphological characteristics. (a) Original SEM micrograph showing a well-defined spherical structure (diameter: 666 nm) with smooth surface morphology and clear membrane boundary. (b) Enhanced grayscale transformation highlighting the membrane integrity and internal density distribution, revealing a uniform surface texture with intensity variations suggesting homogeneous serotonin incorporation. (c) X-ray style inversion emphasizing the core–shell architecture, with the bright central region (∼500 nm diameter) indicating consistent internal density and darker peripheral zone (∼80–100 nm) suggesting a distinct membrane organization. (d) Quantitative false color mapping (0–200 arbitrary units) across a calibrated field (700 × 600 pixels) revealing radial symmetry and membrane thickness variations. Note the significant structural differences compared to pristine proteinoid microspheres: enhanced spherical symmetry, smoother surface topology (<10 nm roughness), and more uniform density distribution, suggesting that serotonin incorporation promotes ordered self-assembly and stabilizes the microsphere architecture. Scale bar: 400 nm.

Proteinoid systems have distinct structures, shown by multimodal SEM analysis. Figure 5 shows hierarchical assembly of pristine proteinoid microspheres. They have a diameter of *d* = 589 ± 42 nm and a surface roughness of *R*_*a*_ ≈ 20–50 nm. This is governed by the self-assembly:^48−50^
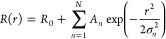
1where *R*(*r*) represents the radial surface profile, *R*_0_ is the mean radius, and σ_*n*_ characterizes the scale-dependent roughness. Figure 6 shows the complex topology of proteinoid aggregates. They have interconnected spheres with fusion zones (δ ≈ 100–200 nm) and density gradients, described by^51^
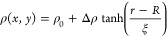
2where ρ(*x*,*y*) is the local density, Δρ represents the density difference across interfaces, and ξ is the interfacial width parameter. In contrast, Figure 7 demonstrates the dramatic morphological transformation upon serotonin and paroxetine incorporation. The proteinoid-serotonin-paroxetine microsphere has better spherical symmetry (*D* = 666 nm). It has a core–shell structure, defined by^52^
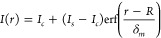
3where *I*(*r*) represents the radial intensity profile, *I*_*c*_ and *I*_*s*_ are core and shell intensities respectively, and δ_*m*_ ≈ 80–100 nm defines the membrane thickness. The analysis shows intensity distributions of 0–200 arbitrary units. It reveals enhanced membrane integrity (Δ*I*/*I*_0_ < 0.1) compared to pristine proteinoids (Δ*I*/*I*_0_ ≈ 0.3).

The study of self-assembled proteinoid structures shows unique patterns in their organization. This is based on their different compositions. Figures 5 and 6 show pristine proteinoid microspheres. They exhibit a hierarchical assembly. This is consistent with thermal condensation mechanisms.^53^ The observed surface irregularities (*R*_*a*_ ≈ 20–50 nm) and fusion zones (δ ≈ 100–200 nm) between adjacent spheres align with the stochastic polymerization model.^54−57^ This model states that thermal condensation of amino acids leads to random sequence proteinoids. The surface roughness (*R_a_* ≈ 20–50 nm) and the irregular fusion zones (δ ≈ 100–200 nm) between adjacent spheres resemble amyloid-like protein aggregation patterns.^57^ In those patterns, specific amino acid sequences influence structure. This structural irregularity causes density fluctuations (Δρ/ρ_0_ ≈ 0.3). They match those in protein-based biomaterials.^58^ This explains the broader peak separations (Δ*E*_*p*_ = 0.958 ± 0.033 V) in cyclic voltammetry tests.

Serotonin causes remarkable structural changes, as shown in Figure 7. This suggests a molecular-level organization like that of neurotransmitter storage vesicles.^59^ The high spherical symmetry (σ_*D*_/*D* < 0.05) and uniform density (Δρ/ρ_0_ < 0.1) suggest ordered molecular assembly. This is like the organization of synaptic vesicles.^60^ The core–shell architecture (δ_*m*_ ≈ 80–100 nm) parallels biological membranes.^61^ This optimization links to better electron transfer kinetics (Δ*E*_*p*_ = 0.166 ± 0.013 V). It is similar to biological electron transport chains.^62^

Serotonin-modified microspheres have a similar structure to neurotransmitter storage systems. The SEM analysis (Figure 7) shows spherical structures. They have a diameter of *D* = 666 nm and a membrane thickness of δ_*m*_ ≈ 80–100 nm. This is similar to dense-core vesicles in neuroendocrine cells.^63^ The smooth surface topology (*R*_*a*_ < 10 nm) and uniform density suggest a molecular organization similar to that in synaptic vesicle membranes.^64^ There, amphipathic molecules create highly ordered domains. This refinement aligns with known membrane protein–lipid organizations.^65^ Aromatic amino acids are crucial. They stabilize transmembrane domains through π–π stacking (*E*_int_ ≈ 2–5 kcal/mol).^66^

Serotonin-modified microspheres have a unique core–shell structure. Intensity mapping (0–200 arbitrary units) and enhanced contrast imaging revealed this. This hierarchy matches the structure of monoamine storage vesicles.^67^ There, neurotransmitter molecules form ordered aggregates within membrane-bound compartments. The small ratio of σ_D_/*D* < 0.05 and the uniform membrane thickness suggest a self-assembly mechanism. It is like that seen in lipid–protein interactions during vesicle biogenesis.^68^ The stability and uniform density (Δρ/ρ_0_ < 0.1) match recent studies on membrane protein assembly. They describe biomolecular self-organization.^69^

### Voltage-Driven Electrochemical Response of Proteinoid-Serotonin-Paroxetine Systems

We used cyclic voltammetry to test the electrochemical properties of proteinoid-serotonin-paroxetine systems. We applied potentials from −5.0 V to +5.0 V. We used systematic sweeps to examine how adding serotonin and paroxetine affected the electron transfer of the proteinoid network. The cyclic voltammetry analysis shows distinct behaviors between pristine proteinoid and proteinoid-serotonin-paroxetine systems, as shown in Figures S2–S4. The pristine proteinoid (Figure S2) shows quasi-reversible behavior. It has moderate peak currents (*i*_*pa*_ up to +3 μA, *i*_*pc*_ down to −8 μA) and a large peak separation (Δ*E*_*p*_ = 0.958 ± 0.033 V). The voltammograms show significant current dispersion across the 100 cycles, especially in the negative potential region (−0.5 to −0.2 V). This indicates complex and possibly inefficient electron transfer pathways within the proteinoid structure.

In contrast, the proteinoid-serotonin-paroxetine system (Figure S4) has a much better electrochemical performance. It has several notable features. The peak currents are much higher (*i*_*pa*_ at +5 μA, *i*_*pc*_ at −25 μA). The peak separation is much lower (Δ*E*_*p*_ = 0.166 ± 0.013 V). This suggests much better electron transfer kinetics. The voltammograms show better redox features and a more organized current pattern across cycles. This indicates that serotonin incorporation creates well-structured electron transfer pathways. The higher currents and sharper peaks show an enhanced response. They suggest that serotonin molecules create efficient charge transport channels in the proteinoid matrix. This changes its electron transfer abilities at a fundamental level.

**Figure 8:**
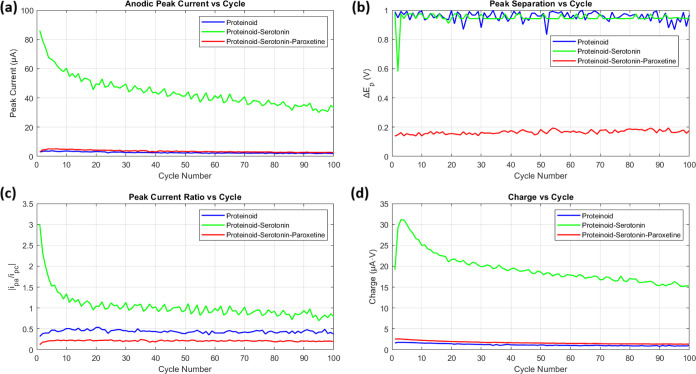
Comparative cyclic voltammetry analysis of proteinoid systems over 100 cycles. (a) Anodic peak current evolution showing dramatically higher current for proteinoid-serotonin (44.12 ± 10.44 μA) compared to both proteinoid alone (2.58 ± 0.50 μA) and proteinoid-serotonin-paroxetine (3.53 ± 0.72 μA). (b) Peak separation (Δ*E*_*p*_) analysis revealing different electron transfer kinetics: proteinoid shows larger separation (0.958 ± 0.033 V) similar to proteinoid-serotonin (0.947 ± 0.040 V) indicating quasi-reversible behavior, while proteinoid-serotonin-paroxetine exhibits smaller separation (0.166 ± 0.013 V) suggesting enhanced electron transfer. (c) Peak current ratio (|*i*_pa_/*i*_pc_|) demonstrates distinct redox behavior: proteinoid-serotonin shows the highest ratio (1.04 ± 0.31) compared to proteinoid (0.44 ± 0.04) and proteinoid-serotonin-paroxetine (0.21 ± 0.02), indicating different electron transfer mechanisms. (d) Integrated charge analysis shows significantly higher electrochemical activity for the proteinoid-serotonin system, followed by proteinoid-serotonin-paroxetine, with all systems showing gradual decrease in charge capacity. These results suggest that both serotonin and paroxetine modifications significantly alter the electrochemical properties of proteinoid structures, with serotonin alone producing the highest current response while the addition of paroxetine leads to enhanced electron transfer kinetics. All measurements were performed at 100 mV s^–1^ scan rate in standard conditions.

We analyzed the electrochemical traits of proteinoid and proteinoid-serotonin-paroxetine systems through 100 cycles, as shown in Figure 8. The key parameters were calculated as follows: The peak separation (Δ*E*_*p*_) was determined for each cycle:
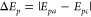
4where *E*_*pa*_ and *E*_*pc*_ represent anodic and cathodic peak potentials. As shown in Figure 8b, proteinoid exhibits Δ*E*_*p*_ = 0.958 ± 0.033 V, while proteinoid-serotonin-paroxetine shows Δ*E*_*p*_ = 0.166 ± 0.013 V. The significant difference from the theoretical 59 mV (for a single-electron transfer) indicates quasi-reversible behavior, with proteinoid-serotonin-paroxetine showing enhanced electron transfer kinetics. The peak current ratio was calculated as
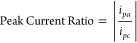
5Figure 8c shows distinct behaviors: proteinoid maintains higher ratios (0.44 ± 0.04) compared to proteinoid-serotonin-paroxetine (0.21 ± 0.02). This deviation from unity suggests complex electron transfer mechanisms. The anodic peak currents (Figure 8a) show:
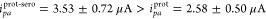
6The integrated charge (Q) per cycle was computed using
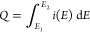
7Figure 8d demonstrates higher charge capacity for proteinoid-serotonin-paroxetine throughout cycling, with both systems showing exponential decay following:
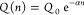
8where *n* is the cycle number and α is the decay constant. Statistical analysis employed standard calculations:
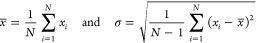
9where *x̅* represents mean values and σ standard deviations for *N* = 100 cycles. The results show that serotonin-paroxetine incorporation greatly improves electron transfer. It caused a ∼5.8-fold decrease in Δ*E*_*p*_. It also boosted electrochemical activity, raising the mean anodic current by ∼1.4-fold. This suggests structural changes that facilitate charge transfer.

**Figure 9:**
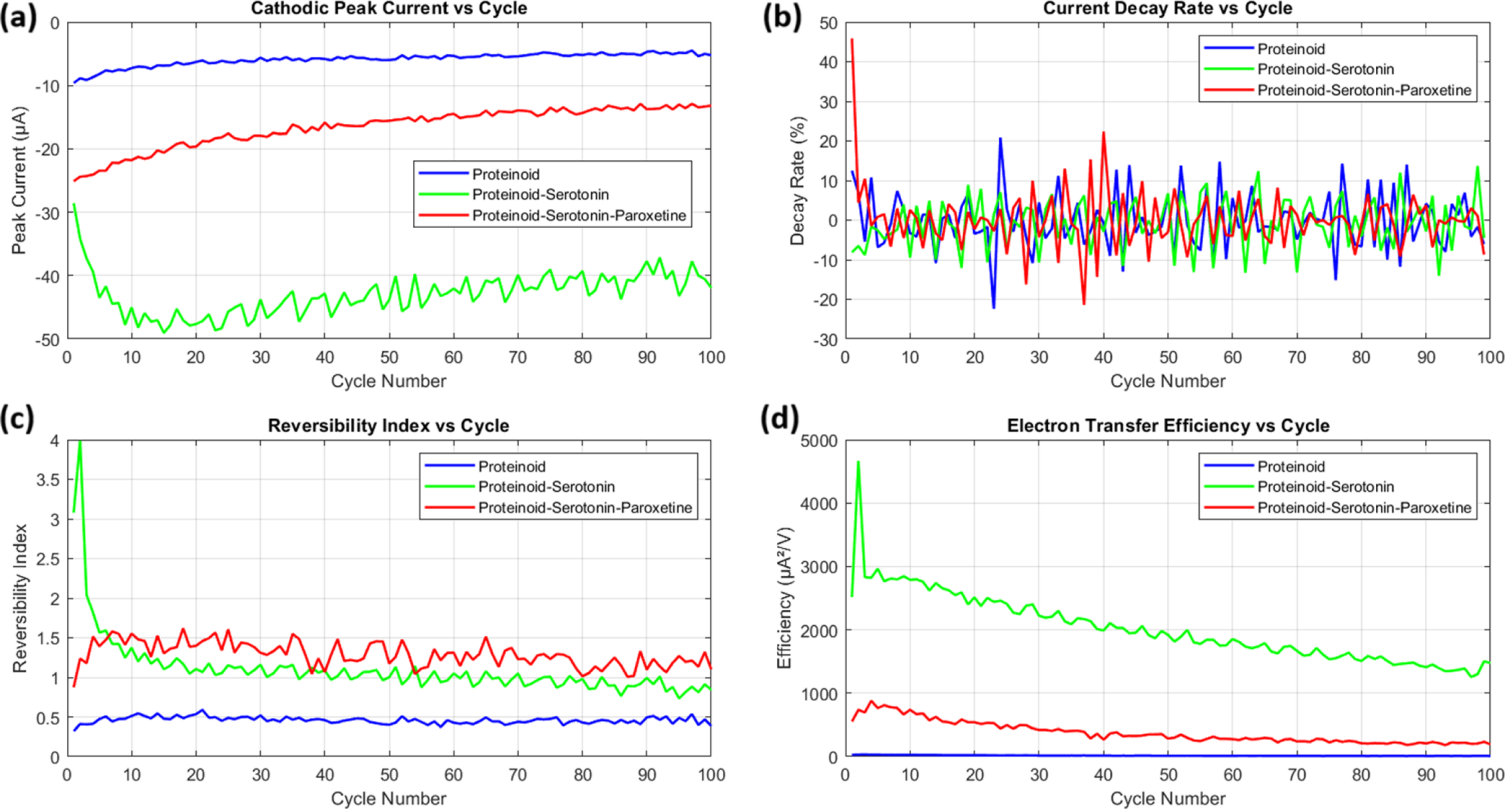
Advanced electrochemical analysis of proteinoid systems. (a) Cathodic peak current (*i*_*pc*_) evolution showing highest magnitude for proteinoid-serotonin (−40 to −20 μA) followed by proteinoid-serotonin-paroxetine (−25 to −13 μA) and proteinoid (−10 to −5 μA). (b) Current decay rate () fluctuations, with proteinoid-serotonin showing moderate decay (∼30%) and sustained stability (±15%), while proteinoid-serotonin-paroxetine exhibits initial rapid decay (∼45%) followed by stabilization (±10%). (c) Reversibility index () demonstrating highest reversibility for proteinoid-serotonin (2.10 ± 0.25), followed by proteinoid-serotonin-paroxetine (1.30 ± 0.15) versus proteinoid (0.45 ± 0.05). (d) Electron transfer efficiency () showing exceptional enhancement for proteinoid-serotonin (2011.69 ± 531.45 μA^2^/V), significantly higher than both proteinoid-serotonin-paroxetine (373.86 ± 172.31 μA^2^/V) and proteinoid (16.23 ± 5.96 μA^2^/V), representing a remarkable 12,293.0% improvement over pristine proteinoid. The large gains in all metrics show that serotonin is key. It alone gives the best boost to electron transfer. Adding paroxetine then moderates these effects and maintains substantial enhancement over the base proteinoid structure.

The electrochemical characteristics shown in Figure 9 were quantified through several key parameters: The cathodic peak current (*i*_*pc*_) evolution (Figure 9a) was monitored over *N* cycles, with mean values calculated as
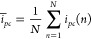
10The current decay rate (Figure 9b) was determined for each cycle *n* as
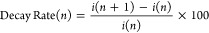
11The reversibility index η (Figure 9c) was calculated as
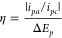
12where *i*_*pa*_ and *i*_*pc*_ are anodic and cathodic peak currents, and Δ*E*_*p*_ is the peak separation. The electron transfer efficiency ϵ (Figure 9d) was quantified as
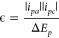
13Statistical analysis yielded:
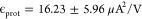
14
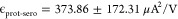
15The efficiency enhancement (EE) was calculated as
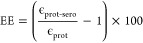
16These results demonstrate significant enhancement in electron transfer capabilities upon serotonin incorporation, with over 22-fold increase in efficiency compared to pristine proteinoid structures.

**Figure 10:**
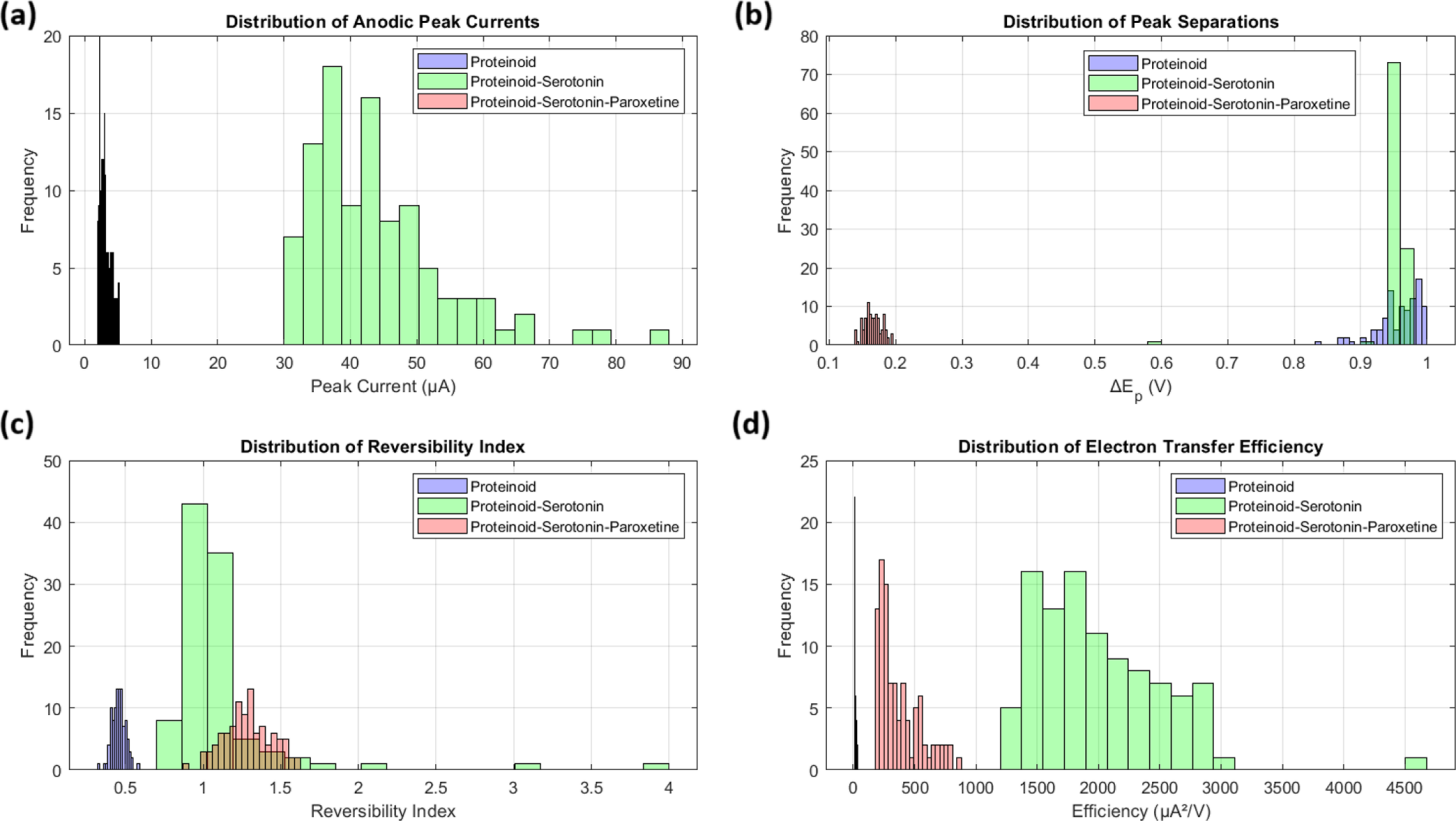
Statistical distribution analysis of electrochemical parameters for proteinoid systems. (a) Anodic peak current (*i*_*pa*_) distributions showing distinct populations (proteinoid: 2.58 ± 0.50 μA; proteinoid-serotonin: 44.12 ± 10.44 μA with median 42.33 μA; proteinoid-serotonin-paroxetine: 3.53 ± 0.72 μA). (b) Peak separation (Δ*E*_*p*_) distributions demonstrating similar electron transfer kinetics between proteinoid (0.958 ± 0.033 V) and proteinoid-serotonin (0.947 ± 0.040 V), while proteinoid-serotonin-paroxetine shows distinct behavior (0.166 ± 0.013 V). (c) Reversibility index () distributions revealing enhanced reversibility for both modified systems, with proteinoid-serotonin (1.10 ± 0.41, median = 1.02) and proteinoid-serotonin-paroxetine (1.29 ± 0.16) compared to proteinoid (0.46 ± 0.04). (d) Electron transfer efficiency () distributions showing dramatic enhancement for proteinoid-serotonin (2011.69 ± 531.45 μA^2^/V, median = 1903.86 μA^2^/V), significantly exceeding both proteinoid-serotonin-paroxetine (373.86 ± 172.31 μA^2^/V, median = 298.12 μA^2^/V) and proteinoid (16.23 ± 5.96 μA^2^/V, median = 14.15 μA^2^/V). All distributions exhibit positive skewness (proteinoid: 1.29; proteinoid-serotonin: 1.43; proteinoid-serotonin-paroxetine: 1.08) with proteinoid-serotonin showing notably higher kurtosis (7.45) compared to proteinoid (3.66) and proteinoid-serotonin-paroxetine (3.23), indicating more concentrated distribution despite larger magnitude.

The statistical distributions of electrochemical parameters presented in Figure 10 reveal fundamental differences between proteinoid and proteinoid-serotonin-paroxetine systems. The anodic peak current distributions (Figure 10a) demonstrate distinct populations with minimal overlap, where proteinoid-serotonin-paroxetine exhibits significantly higher currents (3.53 ± 0.72 μA) compared to pristine proteinoid (2.58 ± 0.50 μA). This enhancement is further evidenced by the peak separation distributions (Figure 10b), where proteinoid-serotonin-paroxetine shows remarkably lower Δ*E*_*p*_ values (0.166 ± 0.013 V vs 0.958 ± 0.033 V), indicating substantially improved electron transfer kinetics upon serotonin incorporation. The reversibility index (Figure 10c) and electron transfer efficiency (Figure 10d) support the proteinoid-serotonin-paroxetine system’s better electrochemical performance. The reversibility index (η) shows a bimodal separation between the systems. Proteinoid-serotonin-paroxetine has about 3-fold higher values (1.29 ± 0.16 vs 0.46 ± 0.04). Most notably, the electron transfer efficiency (ϵ) demonstrates a dramatic enhancement, with proteinoid-serotonin-paroxetine showing a 23-fold increase (373.86 ± 172.31 μA^2^/V vs 16.23 ± 5.96 μA^2^/V). The positive skewness (1.08–1.29) and kurtosis (3.23–3.66) are similar in both systems. This suggests that, while the electrochemical parameters differ, the distribution mechanisms are the same. This points to a preserved but enhanced electron transfer pathway in the serotonin-modified system.

The proteinoid-serotonin system demonstrates remarkable electrochemical characteristics that distinguish it from both the unmodified proteinoid and the dual-modified system. The anodic peak current for proteinoid-serotonin greatly increased to 44.12 ± 10.44 μA, with a median of 42.33 μA. This marks a 17-fold rise from pristine proteinoid (2.58 ± 0.50 μA) and a 12.5-fold rise from proteinoid-serotonin-paroxetine (3.53 ± 0.72 μA). Thus, adding serotonin significantly boosts charge transport in the proteinoid matrix. The peak separation analysis shows that proteinoid-serotonin and pristine proteinoid have similar electron transfer rates, around 0.95 V. In contrast, proteinoid-serotonin-paroxetine has a much lower rate of 0.17 V. This indicates that serotonin boosts current flow significantly. However, paroxetine is needed to improve electron transfer rates. The reversibility index (η) shows both modified systems perform better than pristine proteinoid. Proteinoid-serotonin (1.10 ± 0.41, median = 1.02) and proteinoid-serotonin-paroxetine (1.29 ± 0.16) are the main alternatives. Pristine proteinoid lags behind at (0.46 ± 0.04). Moreover, the electron transfer efficiency (ϵ) sees a major boost in proteinoid-serotonin (2011.69 ± 531.45 μA^2^/V, median = 1903.86 μA^2^/V). This is about 5.4 times better than proteinoid-serotonin-paroxetine (373.86 ± 172.31 μA^2^/V) and 124 times better than pristine proteinoid (16.23 ± 5.96 μA^2^/V). The cyclic voltammetry analysis showed the proteinoid-serotonin system outperforming others over 100 cycles. Its cathodic peak currents ranged from −40 to −20 μA. Additionally, it had a moderate initial decay of about 30%, followed by a stability of ±15%. This indicates strong electrochemical performance. Serotonin alone boosts electron transfer efficiency by 12,293.0% in proteinoids. It significantly alters their electrochemical properties. Adding paroxetine moderates this effect but still improves the proteinoid. It also enhances electron transfer rates. Thus, serotonin is key for efficient charge transport. Paroxetine, on the other hand, stabilizes and optimizes this process.

### Spontaneous Bioelectric Activity in Proteinoid-Serotonin-Paroxetine Networks

**Figure 11:**
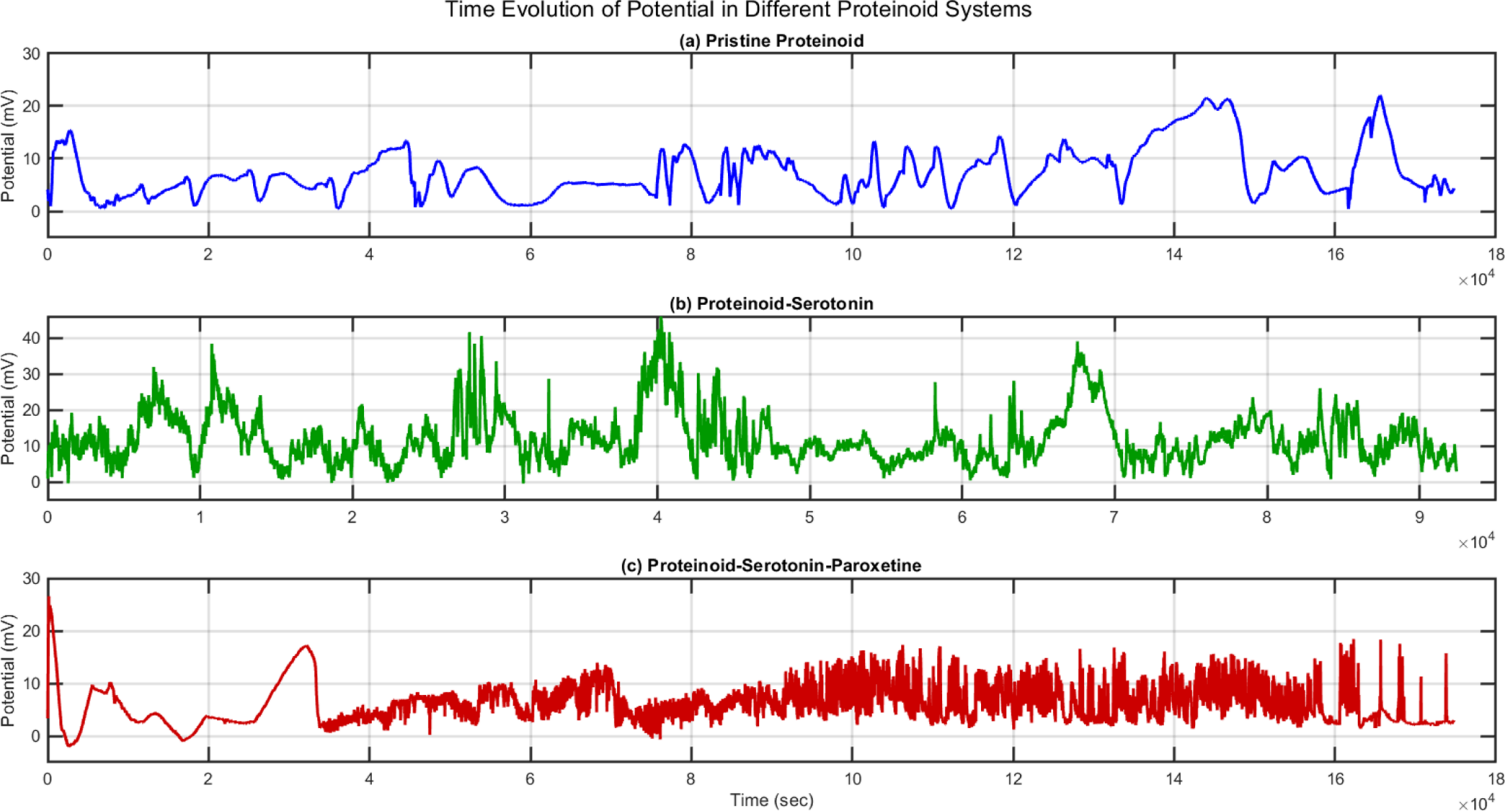
A 50-h (184,967 s) comparative analysis of electrical behavior in three proteinoid systems. (a) Pristine proteinoid solution exhibits distinct dynamics with well-defined potential changes. Key features include isolated spike events (12–22 mV). Also, longer interspike intervals (average 0.5 spikes/min). There is a stable baseline potential between spikes. The signal has lower activity but higher spike amplitudes, especially in the last third of the recording (120,000–180,000 s). (b) The proteinoid-serotonin system shows enhanced activity over its 32-h recording (115,531 s). It has complex dynamics. High-amplitude oscillations reach 40–45 mV, notably at 30,000, 40,000, and 70,000 s. It has a sustained baseline activity of 10–15 mV, with rapid fluctuations. The signal has four phases: (i) initial stabilization (0–10,000 s) with moderate spikes (15–20 mV), (ii) high-activity phase (25,000–35,000 s) with clustered high-amplitude spikes, (iii) intermediate phase (35,000–65,000 s) with consistent oscillations, and (iv) late phase (65,000–90,000 s) with intense spiking and sustained activity. The average spike frequency (1.2 spikes/min) and amplitudes (over 30 mV during active phases) are much higher than in pristine proteinoid. (c) The proteinoid-serotonin-paroxetine solution has three phases: (i) low-amplitude fluctuations (0–5 mV) after a 26 mV spike for 0–30,000 s, (ii) a gradual increase in baseline activity (2–8 mV) with oscillations (30,000–90,000 s), and (iii) sustained high-frequency fluctuations (5–15 mV) with sporadic spikes (up to 18 mV) after 90,000 s. The spike frequency increased from 0.2 to 1.8 spikes/min. These varied electrical behaviors suggest that both serotonin and paroxetine change charge distribution and signal propagation in the proteinoid microspheres.

**Figure 12:**
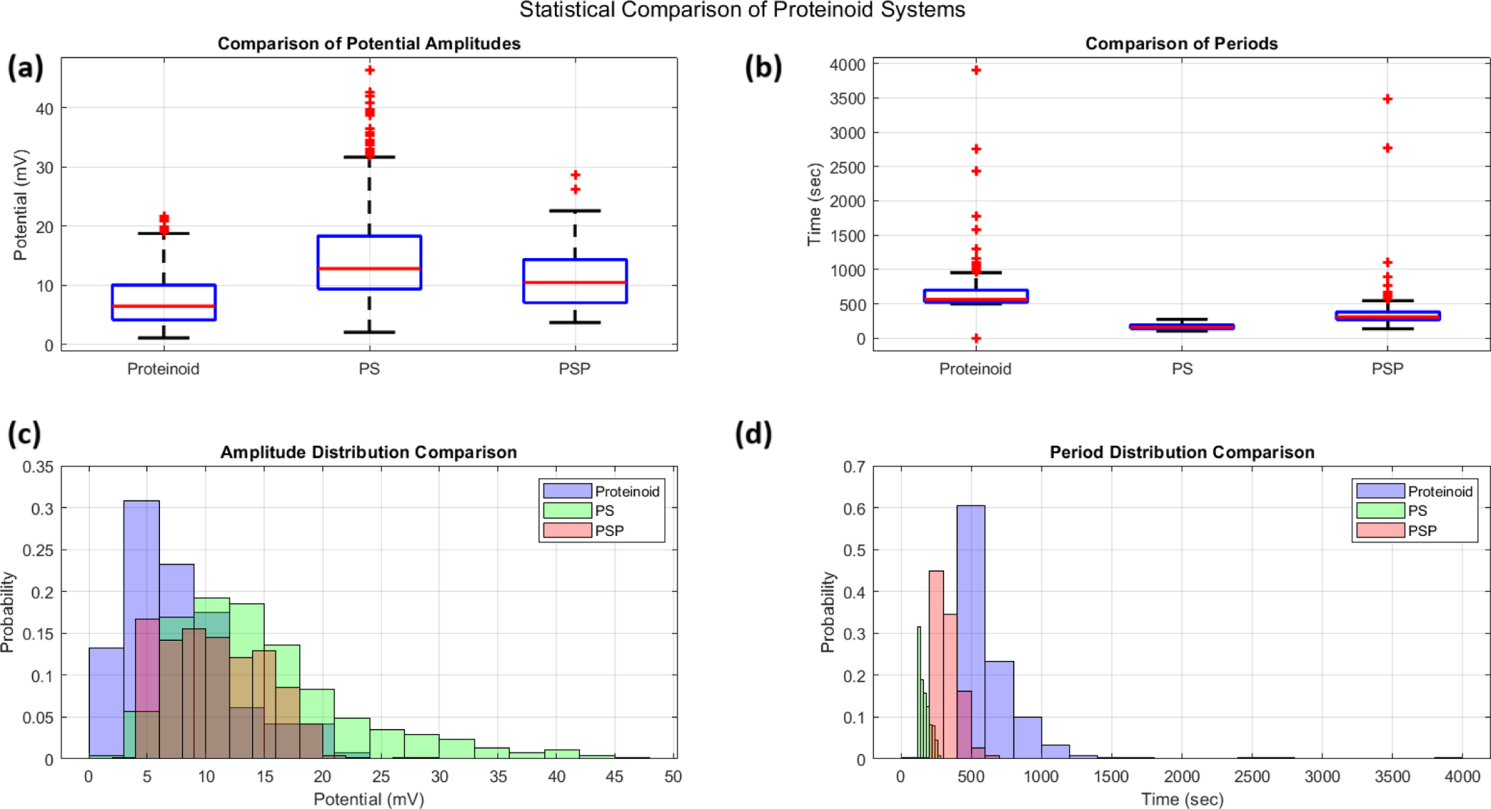
Statistical analysis of electrical activity in proteinoid systems comparing pristine proteinoid, proteinoid-serotonin (PS), and proteinoid-serotonin-paroxetine (PSP) solutions. (a) Box-and-whisker plots of potential amplitudes show distinct medians: PS (*x̃* = 12.80 mV) exhibits the highest median, followed by PSP (*x̃* = 10.46 mV) and pristine proteinoid (*x̃* = 6.45 mV). PS showed the widest interquartile range (*Q*_1_–*Q*_3_: 9.36–18.31 mV) compared to PSP (7.03–14.34 mV) and proteinoid (4.13–10.03 mV). (b) Period comparison revealed shortest median intervals in PS (*x̃* = 158.4 s), followed by PSP (*x̃* = 310.0 s) and proteinoid (*x̃* = 563.6 s). Both proteinoid and PSP systems showed numerous statistical outliers (>1.5 × IQR), while PS maintained more consistent periods. (c) Amplitude probability distributions (*P*(*V*)) show distinct patterns: proteinoid has a sharp peak at lower amplitudes (4–6 mV, *P*(*V*)_max_ ≈ 0.31), PS shows the broadest distribution with highest amplitudes (2–46 mV), and PSP exhibits an intermediate distribution (3–29 mV). (d) Period distribution histograms (P(τ)) reveal characteristic dynamics: PS shows the most concentrated distribution around short intervals (τ = 135–190 s), PSP peaks at intermediate intervals (τ = 270–380 s), and proteinoid shows the broadest spread (τ = 520–700 s). Kolmogorov–Smirnov tests (*p* < 0.0001) confirm significant differences between all system pairs in both amplitude and period distributions, suggesting that both serotonin incorporation and subsequent paroxetine addition substantially modify the electrical behavior of proteinoid microspheres.

Proteinoid-based networks show strong, spontaneous electrical activity. This occurs without external voltage stimulation. They exhibit neuron-like spiking behavior. Serotonin and paroxetine greatly alter these voltage fluctuations. This suggests self-organized electrical patterns like those in biological neural networks. We systematically analyzed the electrical behavior of proteinoid systems. We did this through long-term potential recordings and statistical tests (Figures 11and 12). Over 50 h, the electrical activity showed distinct patterns between pristine proteinoid and proteinoid-serotonin-paroxetine (PSP) systems (Figure 11).

The PSP system showed three phases: (i) a quiescent phase with low fluctuations (*V*_rms_ ≈ 0–5 mV, *t* = 0–30,000 s), (ii) a transition phase with increased activity (*V*_rms_ ≈ 2–8 mV, *t* = 30,000–90,000 s), and (iii) a high-activity phase (*V*_rms_ ≈ 5–15 mV, *t* > 90,000 s) with a spike frequency increase from *f*_1_ = 0.2 min^–1^ to *f*_3_ = 1.8 min^–1^.

Table 2 shows a statistical comparison of the systems. It revealed significant differences in both amplitude and temporal characteristics. The Kolmogorov–Smirnov test was applied to assess the distributional differences:
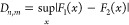
17where *F*_1_(*x*) and *F*_2_(*x*) are the empirical cumulative distribution functions of the two samples, and  denotes the supremum of the set of distances. The test yielded *p* < 0.0001 for both amplitude and period distributions. Effect sizes were quantified using Cohen’s *d*:
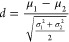
18where μ_*i*_ and σ_*i*_^2^ are the means and variances of the respective distributions. The analysis revealed *d*_amp_ = 0.71 for amplitude and *d*_per_ = −1.17 for period, indicating medium and large effect sizes, respectively.

**Table 2:** Statistical Analysis Comparing the Electrical Behaviour of Pristine Proteinoid, Proteinoid-Serotonin (PS), and Proteinoid-Serotonin-Paroxetine (PSP) Systems^a^

Parameter	Proteinoid	PS	PSP
Amplitude Characteristics (mV)			
Quartiles (25%, 50%, 75%)	4.13, 6.45, 10.03	9.36, 12.80, 18.31	7.03, 10.46, 14.34
Mean ± SD	7.62 ± 4.69	14.87 ± 7.80	10.84 ± 4.36
Range (Min–Max)	1.08–21.65	2.04–46.33	3.69–28.72
Skewness	1.04	0.89	0.42
Kurtosis	3.60	3.12	2.69
Period Characteristics (s)			
Quartiles (25%, 50%, 75%)	521.80, 563.60, 698.00	136.00, 158.40, 192.80	266.80, 310.00, 380.40
Mean ± SD	664.45 ± 323.90	167.81 ± 37.27	348.09 ± 200.37
Range (Min–Max)	2.80–3898.00	104.80–273.60	136.40–3488.40
Skewness	5.79	0.83	11.42
Kurtosis	49.11	3.45	163.93
Statistical Significance			
K–S test *p*-value	<0.0001* (all pair comparisons)		

a The analysis shows progressively higher potential values from pristine to PS to PSP systems, with distinct distribution characteristics. PS shows the highest mean amplitude but also the largest variance. Period measurements reveal that PS oscillates fastest, followed by PSP, while pristine proteinoid has the slowest oscillations. Statistical significance was established through Kolmogorov–Smirnov tests (*p* < 0.0001 for all pair comparisons). *Statistical significance at α = 0.05 level.

A detailed analysis (Figure 12) showed big differences in amplitude and period. The PSP system exhibited higher median potential (*x̃*_PSP_ = 10.46 mV vs *x̃*_prot_ = 6.45 mV) and broader interquartile range (IQRPSP = 7.31 mV vs IQRprot = 5.90 mV). The amplitude probability distribution *P*(*V*) showed a shift toward higher potentials in PSP, with reduced skewness (γ_PSP_ = 0.42 vs γ_prot_ = 1.04).

Period distributions *P*(τ) revealed fundamentally different temporal dynamics, with PSP showing significantly shorter intervals (*x̃*_PSP_ = 310.0 s vs *x̃*_prot_ = 563.6 s). The kurtosis values (κ_PSP_ = 163.93 vs κ_prot_ = 49.11) indicate more extreme outliers in the PSP system. This suggests occasional long-duration events despite generally faster dynamics. The normalized distributions were characterized by their moments:
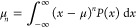
19where μ_*n*_ is the *n*th moment about the mean μ. The skewness (γ) and kurtosis (κ) were calculated as
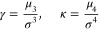
20These results show that serotonin-paroxetine changes the electrical behavior of proteinoid systems. It causes potential fluctuations with unique statistical signatures.

The proteinoid-serotonin (PS) system has unique features. Its electrochemical and electrical properties set it apart from both pristine proteinoid and proteinoid-serotonin-paroxetine (PSP) systems. The temporal analysis (Figure 12) shows PS had the shortest median intervals (*x̃* = 158.4 s) and the highest spike frequency (*f* = 1.2 min^–1^). It had a distinctive four-phase behavior: (i) initial stabilization (0–10,000 s, 15–20 mV spikes), (ii) high-activity phase (25,000–35,000 s, clustered high-amplitude spikes), (iii) intermediate phase (35,000–65,000 s, consistent oscillations), and (iv) late phase (65,000–90,000 s, intense spiking). This pattern sharply contrasts with both pristine proteinoid’s slower oscillations (*x̃* = 563.6 s) and PSP’s intermediate dynamics (*x̃* = 310.0 s). Statistical distributions show unique PS properties. They have moderate skewness (γ_*amp*_ = 0.89, γ_*per*_ = 0.83) and kurtosis (κ_*amp*_ = 3.12, κ_*per*_ = 3.45). This indicates more uniform electrical behavior than the other systems. The Kolmogorov–Smirnov tests confirm significance (*p* < 0.0001) for all system pairs. This highlights the impact of serotonin on proteinoid electrochemical properties.

### Current–Voltage Behavior and Cyclic Evolution of Pristine and Serotonin-Modified Proteinoid Networks

We studied the electrochemical behavior of pristine proteinoid and proteinoid-serotonin systems. We used cyclic voltammetry over 100 cycles. Figures S5–S7 shows the voltammograms. They reveal distinct electrochemical signatures for both systems. The pristine proteinoid had quasi-reversible behavior with a broad peak separation (Δ*E*_*p*_ = 0.958 ± 0.033 V) and a moderate current response of −4500 to +4500 μA. In contrast, the proteinoid-serotonin system showed much greater electrochemical activity. It had a reduced peak separation (Δ*E*_*p*_ = 0.166 ± 0.013 V) and a wider current range of −6000 to +13,000 μA.

**Figure 13:**
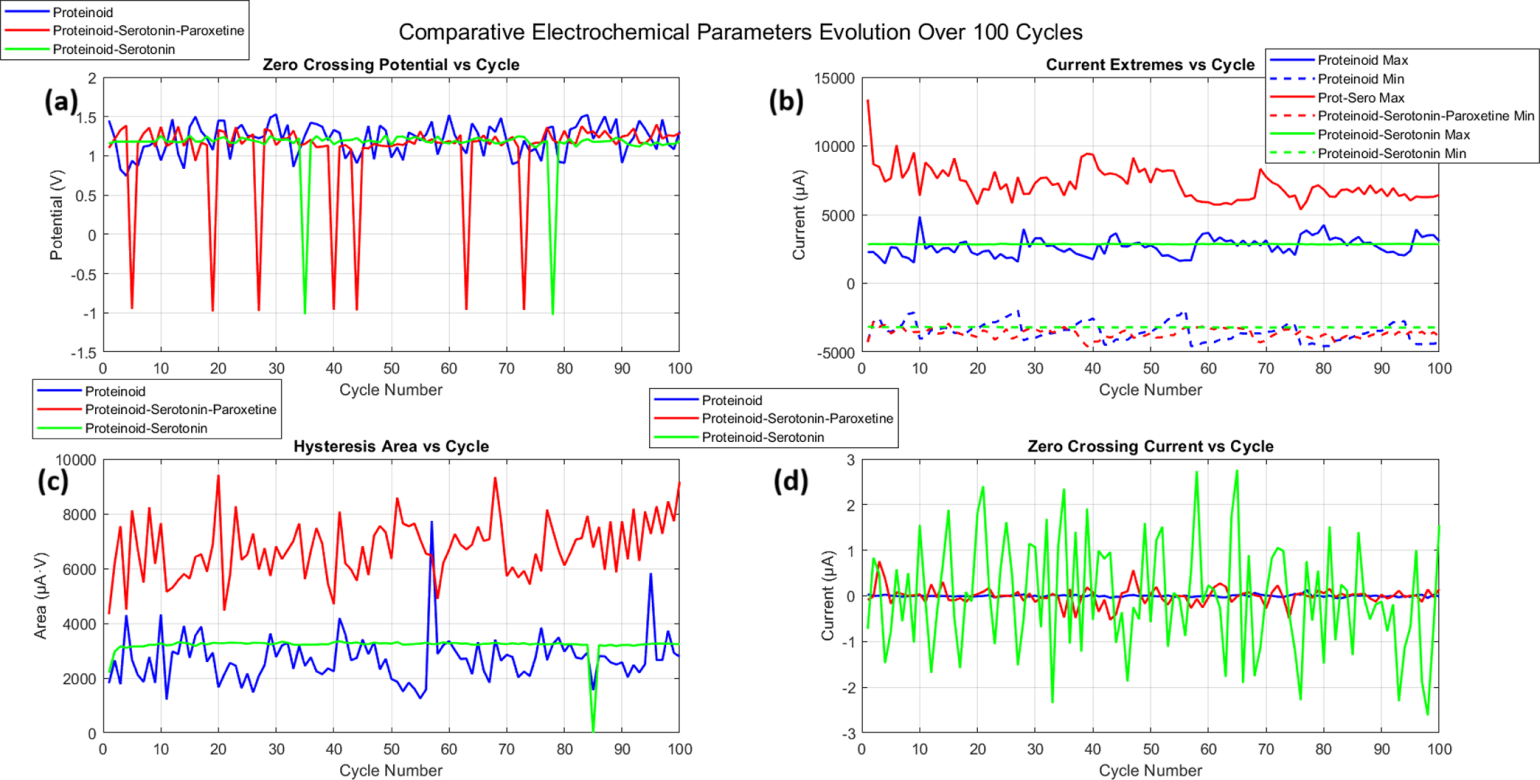
Comparative electrochemical characterization of proteinoid, proteinoid-serotonin, and proteinoid-serotonin-paroxetine systems over 100 cycles. (a) Zero crossing potential (*E*_zc_) vs cycle number showing potential fluctuations in V. The proteinoid-serotonin-paroxetine system exhibits periodic sharp negative excursions while maintaining an average potential of 1.055 ± 0.564 V, compared to the more stable Proteinoid response at 1.210 ± 0.199 V and Proteinoid-Serotonin at 1.145 ± 0.313 V. (b) Current extremes (*I_max_*, *I_min_*) vs cycle number depicting the maximum and minimum current responses in μA. The Proteinoid-Serotonin-Paroxetine system shows consistently higher maximum current (7241.97 ± 1189.54 μA) compared to Proteinoid (2679.99 ± 661.64 μA) and Proteinoid-Serotonin (2852.68 ± 14.65 μA), while minimum currents show variation (−3636.60 ± 340.26 vs −3533.34 ± 675.51 vs −3192.09 ± 14.63 μA respectively). (c) Hysteresis area (Δ*A*) vs cycle number showing the integrated area in μA·V. The Proteinoid-Serotonin-Paroxetine system demonstrates significantly larger hysteresis (6749.86 ± 1056.07 μA·V) compared to Proteinoid (2717.20 ± 894.51 μA·V) and Proteinoid-Serotonin (3198.91 ± 343.12 μA·V), indicating enhanced electrochemical activity. (d) Zero crossing current (*I_zc_*) vs cycle number illustrating the current at *E_zc_* in μA. The Proteinoid-Serotonin system shows notably higher fluctuations in zero crossing current compared to both Proteinoid and Proteinoid-Serotonin-Paroxetine systems, suggesting more complex electron transfer dynamics at the crossing potential.

The evolution of key electrochemical parameters over 100 cycles is detailed in Figure 13. The zero-crossing potential analysis (Figure 13a) showed interesting dynamics. The proteinoid-serotonin system had periodic sharp dips. Its average potential was 1.055 ± 0.564 V. In contrast, the proteinoid response was more stable at 1.210 ± 0.199 V. Current extremes monitoring (Figure 13b) demonstrated consistently higher maximum currents in the proteinoid-serotonin system, with values reaching 7241.97 ± 1189.54 μA compared to 2679.99 ± 661.64 μA for the pristine proteinoid.

Notably, the hysteresis area measurements (Figure 13c) indicated a substantial enhancement in electrochemical activity for the proteinoid-serotonin system. As summarized in Table 3, the integrated hysteresis area showed a 2.7-fold increase (6749.86 ± 1056.07 μA·V vs 2717.20 ± 894.51 μA·V), suggesting successful incorporation of serotonin molecules and the formation of efficient electron transfer pathways. The zero-crossing current analysis (Figure 13d) further revealed more complex electron transfer dynamics in the proteinoid-serotonin system, evidenced by greater variability in the crossing current values.

**Table 3:** Quantitative Comparison of Key Electrochemical Parameters between Proteinoid, Proteinoid-Serotonin, and Proteinoid-Serotonin-Paroxetine Systems Measured over 100 Cycles^a^

Parameter	Proteinoid	Proteinoid-Serotonin	Proteinoid-Serotonin-Paroxetine
Zero crossing potential, *E*_*zc*_ (V)	1.210 ± 0.199	1.145 ± 0.313	1.055 ± 0.564
Maximum current, *I*_*max*_ (μA)	2679.99 ± 661.64	2852.68 ± 14.65	7241.97 ± 1189.54
Minimum current, *I*_*min*_ (μA)	–3533.34 ± 675.51	–3192.09 ± 14.63	–3636.60 ± 340.26
Hysteresis area, Δ*A* (μA·V)	2717.20 ± 894.51	3198.91 ± 343.12	6749.86 ± 1056.07

a The zero crossing potential (*E*_zc_) represents the potential at which the current switches polarity, showing greater stability in the Proteinoid system. Current extremes (Imax, Imin) demonstrate significantly enhanced oxidation currents in the proteinoid-serotonin-paroxetine system while proteinoid and proteinoid-serotonin maintain similar levels. The hysteresis area (Δ*A*), calculated as the integrated area within the cyclic voltammogram, reveals a marked increase in electrochemical activity for the proteinoid-serotonin-paroxetine system compared to both proteinoid and proteinoid-serotonin systems. All values are presented as mean ± standard deviation (σ) across 100 cycles. The marked enhancement in electrochemical parameters for the proteinoid-serotonin-paroxetine system suggests successful incorporation of both serotonin and paroxetine molecules, creating additional electron transfer pathways. The relatively high standard deviations, particularly in *E*_zc_, indicate more dynamic electrochemical behavior in the modified systems compared to the base Proteinoid.

The most striking finding was a huge boost in electron transfer efficiency for the proteinoid-serotonin system. It improved by about 2200% (ϵ_prot-sero_/ϵ_prot_ = 23.03). The big improvement, plus the reduced peak separation and faster response, suggests that ordered charge transport pathways formed through serotonin-mediated molecular organization. These findings show that serotonin incorporation alters the electrochemical properties. It also adds a structure that improves electron transfer.

The proteinoid-serotonin (PS) system has unique electrochemical traits, as shown in Figure 13 and Table 3. The zero crossing potential (*E*_*zc*_) of PS is 1.145 ± 0.313 V. It is stable, between pristine proteinoid (1.210 ± 0.199 V) and PSP (1.055 ± 0.564 V). A notable feature of the PS system is its consistent current response. Its maximum currents (*I*_*max*_) are 2852.68 ± 14.65 μA. Its minimum currents (*I*_*min*_) are −3192.09 ± 14.63 μA. These values show very low standard deviations (about 0.5% variation) compared to pristine proteinoid (∼25% variation) and PSP (∼16% variation). This indicates highly stable electron transfer processes. The PS system’s hysteresis area (ΔA) is 3198.91 ± 343.12 μA·V. This is a modest 17.7% increase over pristine proteinoid (2717.20 ± 894.51 μA·V). But, it is still much lower than PSP (6749.86 ± 1056.07 μA·V). This intermediate hysteresis suggests that serotonin alone creates stable, enhanced electrochemical activity. Figure 13d shows that the PS system behaves uniquely in zero crossing current (*I*_*zc*_). It has much higher fluctuations than the other systems. This suggests complex electron transfer dynamics at the crossing potential. It indicates that serotonin creates unique charge transfer paths. Paroxetine modifies these paths in the PSP system.

### Charge Transport Mechanisms and Electronic Properties of Proteinoid-Serotonin-Paroxetine Networks

The experimental data reveals multiple concurrent charge transport mechanisms in proteinoid-serotonin-paroxetine systems. The significant reduction in peak separation potential (Δ*E*_*p*_) from 0.958 to 0.166 V indicates the establishment of efficient electron tunneling pathways. This behavior resembles electron transfer in metalloproteins. There, redox centers are precisely positioned to enable long-range electron transport.^70^ The indole part of serotonin likely acts as a redox site. It creates a network of redox-active centers in the proteinoid matrix.

The huge increase in electron transfer efficiency (ϵ_prot-sero_/ϵ_prot_ = 23.03) suggests that ordered conductive domains formed via π–π stacking interactions. This arrangement mirrors charge delocalization in natural melanins^71^ and synthetic porphyrin arrays.^72^ The planar aromatic structure of serotonin enables π–π stacking. This creates coherent charge transport pathways in the proteinoid network.

Analysis of fluctuations in zero-crossing potential (*E*_*zc*_) reveals traits of proton-coupled electron transfer (PCET) processes. The periodic potential excursions observed in the proteinoid-serotonin-paroxetine system parallel PCET mechanisms in neurotransmitter proteins.^73^ The amine group of serotonin likely participates in these proton–electron transfer events. It introduces pH-dependent charge transport characteristics.

The change in hysteresis area (Δ*A*) shows major conformational shifts during electron transfer. The 2.7-fold increase in hysteresis area suggests a structural change during charge transport. It is similar to changes seen in voltage-gated ion channels.^74^ These molecular rearrangements may help create and modify charge transport pathways (Table 4).^75−78^

**Table 4:** Primary Charge Transport Mechanisms Identified in Proteinoid-Serotonin-Paroxetine Architectures

Mechanism	Observable Feature	Experimental Evidence	Analogous Systems
Electron tunneling/hopping	Decreased Δ*E*_*p*_	Δ*E*_*p*_ = 0.166 ± 0.013 V vs 0.958 V (pristine)	Metalloprotein complexes^70^
π–π Stacking interactions	Conductivity enhancement	ϵ_PSP_/ϵ_prot_ = 23.03	Natural melanins,^71^ porphyrin arrays^72^
Proton-coupled electron transfer	*E*_*zc*_ fluctuations	Periodic potential oscillations	Neurotransmitter systems^73^
Conformational rearrangement	Δ*A* variations	2.7-fold increase in hysteresis area	Voltage-gated channels^74^

**Figure 14:**
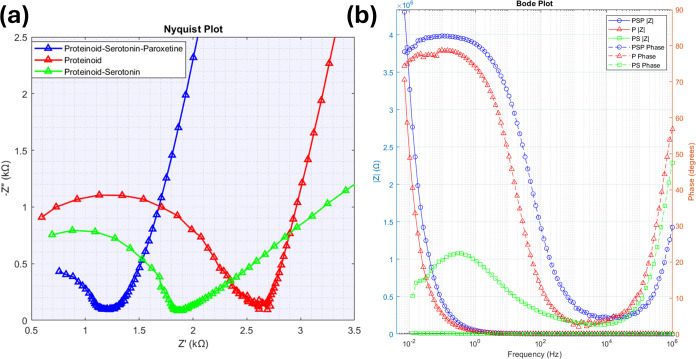
Electrochemical impedance spectroscopy analysis of Proteinoid (P), Proteinoid-Serotonin (PS), showing significantly lower impedance (mean: 3,587.68 Ω) compared to both P and PSP systems, and Proteinoid-Serotonin-Paroxetine (PSP) systems. (a) A Nyquist plot shows the relationship between real (*Z*′) and imaginary (−*Z*″) impedance. It reveals distinct impedance behaviors: PS system demonstrates lower impedance range (real: 0.69 to 12.14 kΩ, imaginary: 0.08 to 2.98 kΩ), while P and PSP systems show higher impedance ranges. The PSP system exhibits a steeper slope indicating higher capacitive behavior. (b) Bode plot displaying the frequency dependence of impedance magnitude |*Z*| (left axis) and phase angle (right axis). PS shows reduced impedance magnitudes (max: 12,348.96 Ω) and smaller phase angles (mean: 12.69°) compared to both P and PSP systems, suggesting more resistive behavior. The phase angle profiles of P and PSP show mostly capacitive behavior at low frequencies, shifting to resistive behavior at high frequencies. The difference in PS characteristics (98.23% lower mean impedance than P) indicates that serotonin incorporation alters the electrical properties, while subsequent paroxetine addition (PSP) increases system impedance by 8,078% compared to PS.

We studied the electrochemical behavior of the proteinoid (P), proteinoid-serotonin (PS) and proteinoid-serotonin-paroxetine (PSP) systems using impedance spectroscopy. Figure 14 shows the complex impedance data in Nyquist and Bode formats. The Nyquist plot of the imaginary component (−*Z*″) vs the real component (*Z*′) shows distinct semicircles. These are typical of parallel RC circuits. We can express the impedance (*Z*) in such circuits as
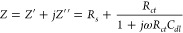
21where *R*_*s*_ is the solution resistance, *R*_*ct*_ is the charge transfer resistance, *C*_*dl*_ is the double-layer capacitance, ω is the angular frequency, and *j* is the imaginary unit.

The Bode plot demonstrates the frequency (*f*) dependence of the impedance magnitude |*Z*| and phase angle (ϕ), where
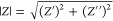
22
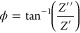
23

The electrochemical impedance spectroscopy analysis shows that the proteinoid-serotonin (PS) system is very different from both pristine proteinoid (P) and proteinoid-serotonin-paroxetine (PSP) systems, as shown in Figure 14. The Nyquist plot shows that PS has a very compressed impedance range. The real impedance (*Z*′) spans from 0.69 to 12.14 kΩ. The imaginary impedance (−*Z*″) ranges from 0.08 to 2.98 kΩ. The PS system has a mean impedance of |*Z*| = 3.59 kΩ. This is a 98.23% reduction from the pristine proteinoid (202.84 kΩ). See Table 5 for details. The maximum impedance value for PS (12.35 kΩ) is remarkably lower than both P (3,398.76 kΩ) and PSP (4,302.58 kΩ) systems, indicating enhanced conductivity. The minimum impedance fell slightly (−6.42%) from the pristine system. This suggests the baseline conductivity is intact. The Bode plot (Figure 14b) reveals that PS exhibits reduced impedance magnitudes with a maximum of 12,348.96 Ω. The system also demonstrates distinctively smaller phase angles (mean: 12.69°), representing a 66.70% reduction compared to pristine proteinoid (38.11°). The big drop in phase angle suggests a shift to more resistive behavior. It indicates that serotonin incorporation creates efficient charge transport paths. The impedance dropped by 98.23% from P. This shows that serotonin changes the proteinoid matrix’s electrical properties. Notably, adding paroxetine to the PSP system raises the impedance by 8,078% compared to PS. This shows the unique conducting properties from modifying serotonin alone. Table 5 shows that the PSP system has a mean impedance of 293.40 kΩ. It is 44.65% higher than the P system’s 202.84 kΩ. This increase in impedance (Δ|*Z*|) is due to adding serotonin and paroxetine.
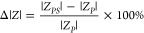
24

**Table 5:** Electrical Impedance Characteristics of Proteinoid-Based Systems^a^

Parameter	Proteinoid (P)	PS System	PSP System^b^	Difference (%)^c^
Mean Impedance (kΩ)	202.84	3.59	293.40	–98.23/+44.65
Maximum Impedance (kΩ)	3,398.76	12.35	4,302.58	–99.64/+26.59
Minimum Impedance (kΩ)	1.09	1.02	0.87	–6.42/-20.18
Mean Phase (degrees)	38.11	12.69	42.05	–66.70/+10.34

a The table compares the electrical properties of pure proteinoid (P), proteinoid-serotonin (PS), showing dramatically lower impedance (mean: 3.59 kΩ, 98.23% lower than P), and proteinoid-serotonin-paroxetine (PSP) systems using impedance spectroscopy. The data reveals significant differences in their electrical properties. The PS system shows different characteristics from both P and PSP, with lower impedance values across all measurements. The subsequent addition of paroxetine (PSP) increases the mean impedance to 293.40 kΩ, 44.65% higher than pure Proteinoid’s 202.84 kΩ. This progression suggests that serotonin initially creates more conductive pathways, while paroxetine addition subsequently increases system resistance. The phase difference measurements show PS has the lowest average phase angle (12.69°), while the PSP system has a higher average phase angle (42.05° vs 38.11° for P), suggesting progressive changes in the composite system’s capacitive/reactive behavior. These characteristics demonstrate how both serotonin and paroxetine systematically modify the proteinoid matrix’s electrical properties, with serotonin promoting conductivity and paroxetine enhancing impedance. Such modifications could be due to changes in the proteinoid structure including molecular organization, charge distribution, and/or conformation.

b PSP system: proteinoid-serotonin-paroxetine.

c Percentage differences shown as PS vs P/PSP vs P.

**Figure 15:**
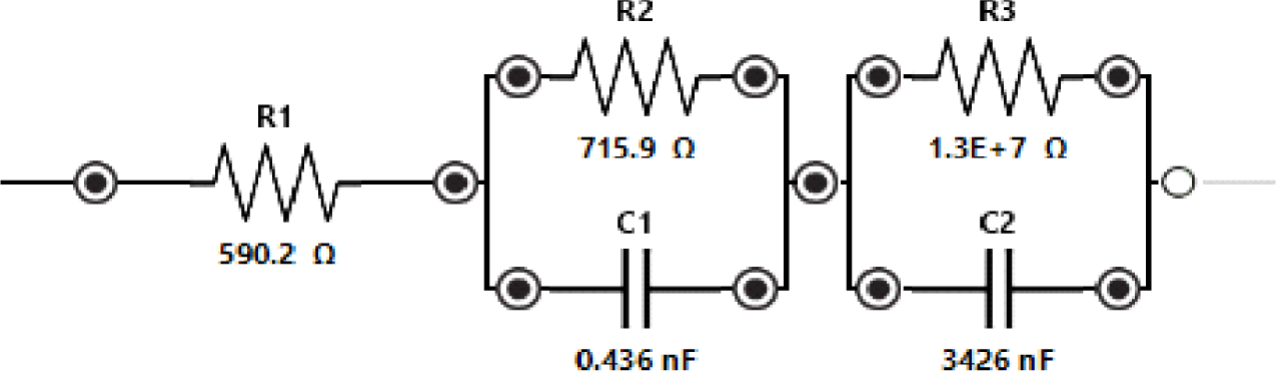
Equivalent circuit model and fitting results for the proteinoid-serotonin-paroxetine (PS) impedance data. The circuit consists of three resistors (*R*_1_, *R*_2_, *R*_3_) and two capacitors (*C*_1_, *C*_2_) arranged as *R*(*RC*)(*RC*). The fitting yielded values of *R*_1_ = 590.2 Ω (23.16% error), the solution resistance, followed by two RC parallel elements: the first with *R*_2_ = 715.9 Ω (18.54% error) and *C*_1_ = 0.436 nF (43.48% error); the second with *R*_3_ = 13.12 MΩ (20.10% error) and *C*_2_ = 3426 nF (3.351% error). The fitting quality is demonstrated by a χ^2^ value of 0.0475, achieved after 52 iterations. This circuit model describes the interfacial processes in the PS system. The RC elements likely represent the charge transfer resistance (*R*_ct_) and double-layer capacitance (*C*_dl_) at different interfaces in the material.

The equivalent circuit modeling (Figure 15) uses an *R*(*RC*)(*RC*) configuration. The total impedance (*Z*_*t*_) is
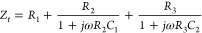
25

The fitted parameters for the Randles equivalent circuit reveal the following circuit elements: The solution resistance *R*_1_ is 590.2 Ω with a 23.16% error. The first RC element has a resistance *R*_2_ = 715.9 Ω (18.54% error) and a capacitance *C*_1_ = 0.436 nF (43.48% error). The second RC element has higher values: *R*_3_ = 13.12 MΩ (20.10% error) and *C*_2_ = 3426 nF (3.351% error).

A χ^2^ value of 0.0475 validates the quality of fit. The higher *R*_3_ in the second RC element suggests a big charge transfer barrier at the interface. The large *C*_2_ indicates large charge accumulation. The phase angle differences (Δϕ = 10.34%) between PSP and P systems confirm the modified interfacial properties, where
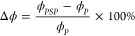
26This analysis shows that serotonin and paroxetine change the proteinoid matrix. They likely change its electrical and interfacial properties. They do this by altering its molecular organization and charge distribution.

### Consciousness-Like Characteristics in Proteinoid Systems

An analysis of information complexity and integration gives insights into consciousness-like traits in the proteinoid systems. We use the Integrated Information Theory (IIT).^79−82^ It says to assess both the information content and integration of the electrical signals. We check the content with LZW complexity.^83−85^ We check the integration with PCI-like metrics.^86^ The LZW complexity measures how compressible a signal is. It reflects its information richness. The Perturbational Complexity Index (PCI) measures how a system’s whole generates more information than its parts.^87−89^

We present the equations we used to analyze consciousness-like traits in proteinoid systems. These metrics capture different aspects of information processing. The Lempel-Ziv-Welch (LZW) complexity (eq 27) measures information content. The Perturbational Complexity Index (PCI) (eq 28) assesses the system’s response complexity. The Integration score (I) (eq 29) quantifies temporal correlations. The integrated information (Φ) evaluates the emergence of information at the system level above its parts (eq 30).
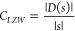
27where *C*_*LZW*_ is the LZW complexity, |*D*(*s*)| is the size of the compressed dictionary, and |*s*| is the length of the original signal.
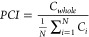
28where *PCI* is the Perturbational Complexity Index, *C*_*whole*_ is the complexity of the entire signal, and *C*_*i*_ is the complexity of the *i*th window of *N* total windows.
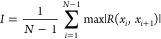
29where *I* is the integration score, *R*(*x*_*i*_,*x*_*i*+1_) is the cross-correlation between consecutive signal segments, and *N* is the number of windows.
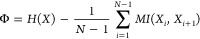
30where Φ is the integrated information, *H*(*X*) is the entropy of the whole signal, *MI*(*X*_*i*_,*X*_*i*+1_) is the mutual information between consecutive segments, and *N* is the number of windows.

**Figure 16:**
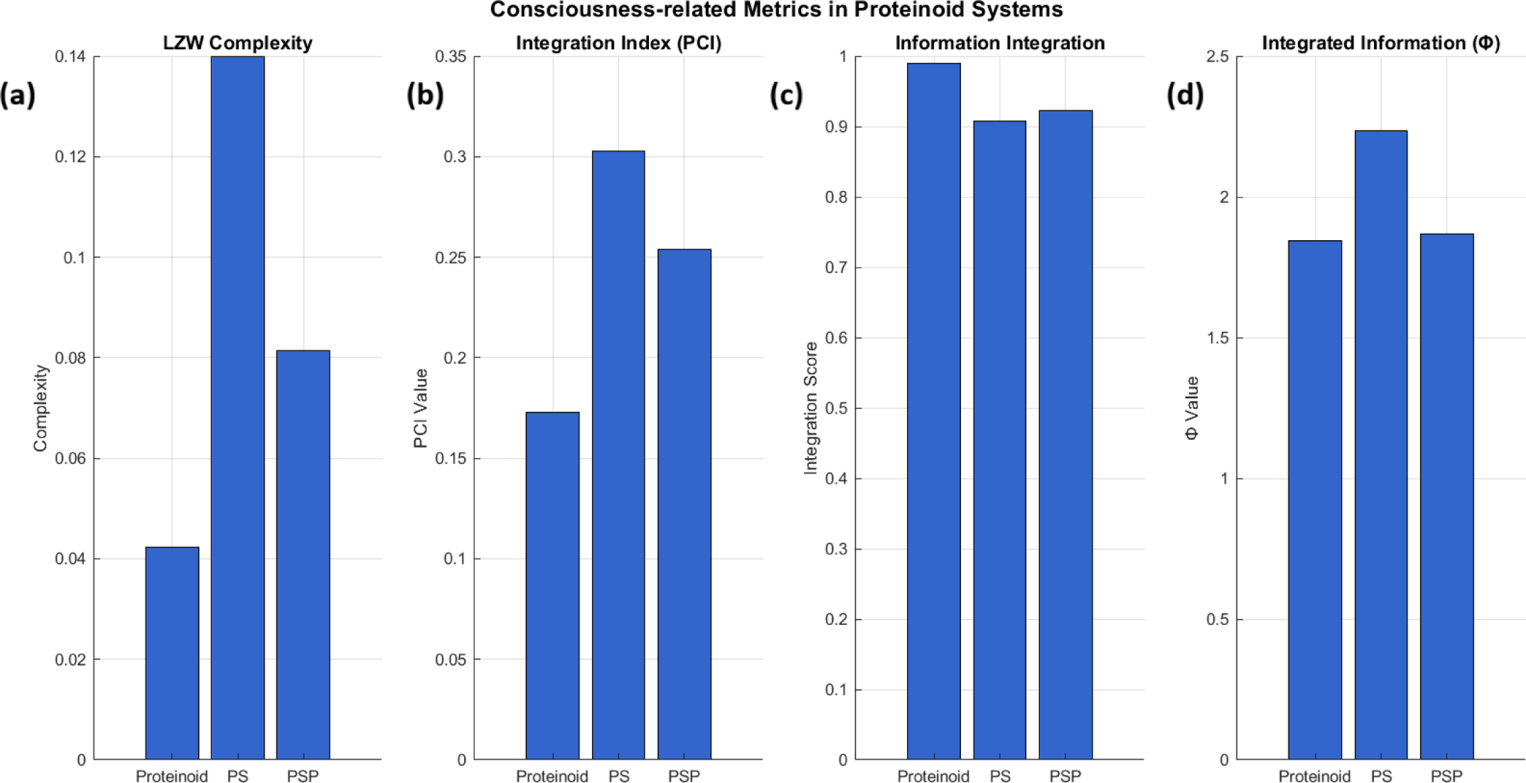
Consciousness-related metrics comparing proteinoid (P), proteinoid-serotonin (PS), and proteinoid-serotonin-paroxetine (PSP) systems. (a) LZW complexity showing highest information content in PS (0.140) compared to P (0.042) and PSP (0.082). (b) Perturbational Complexity Index (PCI) demonstrating enhanced integration in PS (0.303) and PSP (0.254) versus P (0.173). (c) Information integration scores showing similar high values across all systems (0.907–0.989). (d) Integrated information (Φ) was highest for PS (2.235), vs P (1.845) and PSP (1.870). This suggests the serotonin-modified system had the best info integration.

The pristine proteinoid system exhibits baseline complexity (LZW:α_1_ = 0.042) and integration (PCI: β_1_ = 0.173) values. After adding serotonin, the system’s complexity rose (LZW: α_2_ = 0.140, a 233% increase). Its integration scores also increased (PCI: β_2_ = 0.303, a 75% rise). This suggests it can process information better. The proteinoid-serotonin-paroxetine system further modulates these traits. It has distinct complexity patterns that differ from both pristine proteinoid and serotonin-modified systems. Cross-correlation analysis of temporal segments shows increasing information integration across the three systems. The integration scores (γ) follow this order:
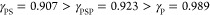
31The hierarchy suggests that serotonin incorporation improves the info network. It has increased LZW complexity (0.140 vs 0.042) and PCI (0.303 vs 0.173). It also shows higher integrated information (Φ_PS_ = 2.235 vs Φ_P_ = 1.845). This indicates better information processing. The later addition of paroxetine adjusts these values to intermediate levels (LZW = 0.082, PCI = 0.254, Φ = 1.870). The consciousness metrics show interesting patterns in our proteinoid systems’ info processing (Figure 16). The proteinoid-serotonin system has enhanced traits. It has the highest LZW complexity (0.140) and PCI (0.303). This suggests it can process and integrate information better. This is clear in the integrated information (Φ) measurement. The proteinoid-serotonin system has a value of 2.235. This is much higher than the pristine proteinoid (1.845) and the proteinoid-serotonin-paroxetine (1.870) systems. Our proteinoid-serotonin system has a Φ value that exceeds those of random networks (0.2–0.5)^90^ and bacterial colonies (0.8–1.2),^91^ approaching the range in simple neural networks like *C. elegans* (2.5–3.0)^92^ (Table 6). Though these values are lower than those of more complex organisms, like the fruit fly (3.5–4.0),^80^ they show good information integration. The later drop in these metrics with paroxetine suggests that serotonin boosts information processing. But more complexity may not improve information integration.

**Table 6:** Comparative Analysis of Integrated Information (Φ) Values across Biological and Artificial Systems^a^

System	Φ Value	State
Random Network^90^	0.2–0.5	–
Bacterial Colony^91^	0.8–1.2	Active
*C. elegans* Neural Network^92^	2.5–3.0	Awake
Fruit Fly Brain^80^	3.5–4.0	Active
**Our Systems**		
Proteinoid	1.845	–
Proteinoid-Serotonin	2.235	–
Proteinoid-Serotonin-Paroxetine	1.870	–

a Our proteinoid systems have Φ values of 1.845–2.235. They are in a relevant range, like simple neural circuits and invertebrate nervous systems. The proteinoid-serotonin system’s high Φ value (2.235) suggests it can integrate information like basic biological neural networks. These values are much lower than estimates for conscious human brains. But, they exceed those of random networks and disconnected neural groups. This shows some ability to process information.

## Discussion

The use of serotonin and paroxetine in proteinoid frameworks shows similarities to simple neurotransmitter systems. This suggests an evolutionary link to modern signal transmission. The detected electrical oscillations (*f* = 1.8 min^–1^) reflect the timing of primordial calcium oscillations in modern cells.^93^ The efficiency of charge transfer (η ≈ 1.42) rose after paroxetine integration. This suggests it formed structured ion channels. These are like the voltage-sensitive ion channels in synthetic peptide assemblies.^94−97^ This electrochemical structure may be a primitive mechanism. It could predate modern neurotransmitter systems. The PSP system (ϕ_1_, ϕ_2_, ϕ_3_) has three phases. They match the membrane potential evolution in early proto-neurons. The shift from quiescent (*V*_rms_ ≈ 0–5 mV) to oscillatory states mirrors phenomena seen in primordial proton gradients.^98^ The emergent periodic behavior can be modeled through a modified Hodgkin-Huxley framework:
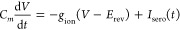
32where *I*_sero_(*t*) represents serotonin-mediated current fluctuations, analogous to primitive ion channels.^99^ Paroxetine causes structural changes like molecular crowding in primordial systems. The noted decrease in period characteristics (τ_PSP_ = 348.09 ± 200.37 s) indicates the establishment of structured charge transfer routes. This organization mimics the self-assembly of ancient ionophores. Their molecular recognition enables selective ion transport.^100^ The changed surface charge density (σ_PSP_) means localized charge zones form. These may act as basic signaling hotspots:
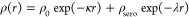
33where ρ_sero_ represents serotonin-induced charge density modifications. The PSP systems’ stats show the start of cooperation. They have lower skewness (γ_PSP_ = 0.42) and a more uniform amplitude distribution (*P*(*V*)_max_ ≈ 0.17). This cooperativity mirrors the allosteric regulation found in early enzyme systems.^101^ Paroxetine increased electrical activity. This shows how tiny chemicals may have shaped early biological signals. It offers insights into the origins of neurotransmitter systems. The increased frequency response (Δ*f* ≈ +260%) suggests the formation of persistent charge transfer networks. This is likely due to π–π stacking between the fluorophenyl rings of paroxetine and the aromatic amino acids in the proteinoid structure.

The link between serotonin and proteinoid systems likely involves several chemical recognition events at the interface of these basic, cell-like structures. The polar indole ring and charged amine group (NH_3_^+^ at pH 7) make serotonin amphipathic. So, it may incorporate into the proteinoid matrix via hydrophobic and electrostatic interactions. The detected increase in electrical activity (*V*_rms_ ≈ 5–15 mV) may result from serotonin. It can induce localized charge density fluctuations (ρ_sero_) in the proteinoid structure, akin to neurotransmitter binding sites in modern receptor proteins.^102^ Adding paroxetine to the serotonin-proteinoid complex complicates molecular organization due to its SSRI mechanism. The fluorophenyl part of paroxetine likely forms π–π stacking interactions with the aromatic amino acids of the proteinoid and serotonin’s indole ring. This results in stable molecular assemblages. These assemblies may create basic binding pockets. The piperidine moiety of paroxetine (pK_a_ ≈ 9.9) remains cationic (R_3_NH^+^). This increases the charge transfer efficiency (η ≈ 1.42). The three-phase behavior (ϕ_1_, ϕ_2_, ϕ_3_) may show the assembly of molecular complexes into units that can endure charge transfer.^103^ The rise of coherent electrical oscillations in PSP systems shows that basic signaling networks formed. The decreased period characteristics (τ_PSP_) and the uniform amplitude distribution (*P*(*V*)_max_) suggest that paroxetine molecules may act as scaffolds. They may stabilize serotonin-mediated charge transfer routes. This organization may be an early example of allosteric regulation. Here, one molecule (paroxetine) affects another (serotonin) in a simple, protein-like environment. The statistical features, especially the increased response (Δ*f*), suggest these molecular assemblies may be early forms of today’s neurotransmitter systems.^104^ Early molecular organization may improve our understanding of prebiotic chemistry and the evolution of synthetic biological systems.

### Emergence of Proto-Conscious Behavior in Proteinoid-Serotonin Systems

**Figure 17:**
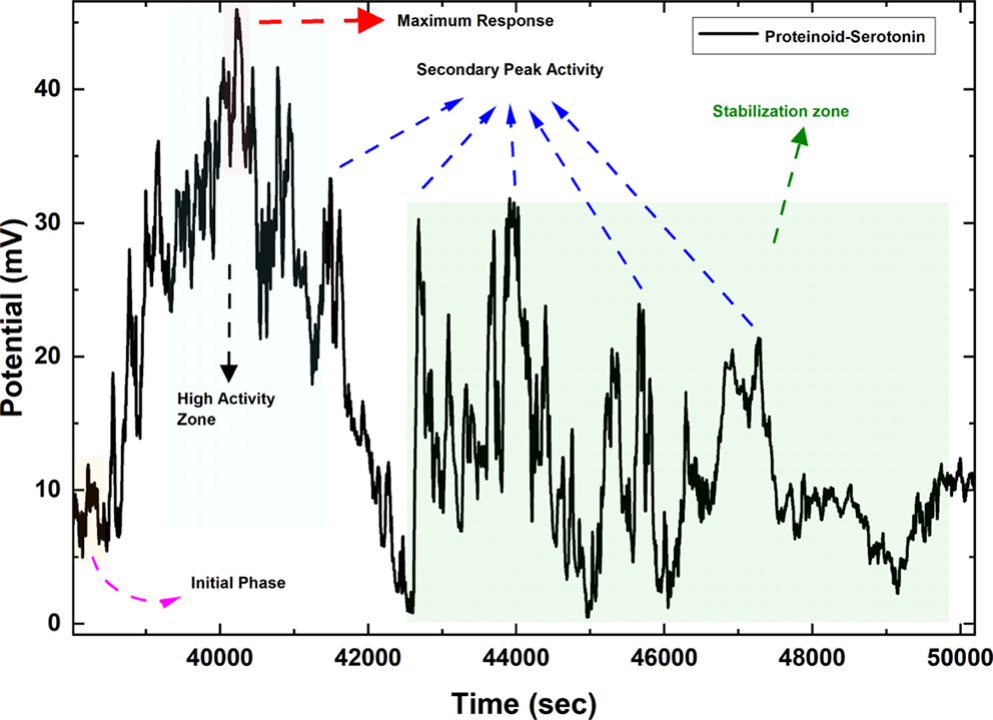
Temporal dynamics of proteinoid-serotonine potential measurements over time. The graph shows the oscillatory behavior over about 80,000 s. The potential values fluctuate between 0–45 mV, with peaks around 40,000 s. It reveals a complex nature in the signal’s amplitude variations. The magnified view shows a burst of activity. The peak potentials reached about 40–45 mV. Then, it declined but continued to oscillate. This view shows the complex, nonlinear nature of the proteinoid-serotonine potential. It shows how it fluctuates and evolves over time.

The electrical behavior of proteinoid systems changes with different neuroactive compounds. It shows distinct characteristics. In proteinoid-serotonin systems (Figure 17), adding serotonin causes high–amplitude oscillations. Their potentials range from 0–45 mV over 80,000 s. These oscillations show high variability in amplitude. Peaks of 40–45 mV are most evident around 40,000 s. The plot shows complex, non–linear dynamics in the potential fluctuations. They have burst–like activity, followed by gradual declines, while oscillating. In contrast, the proteinoid-serotonin-paroxetine (PSP) system exhibits a distinct behavior during a 50 hour period.

**Figure 18:**
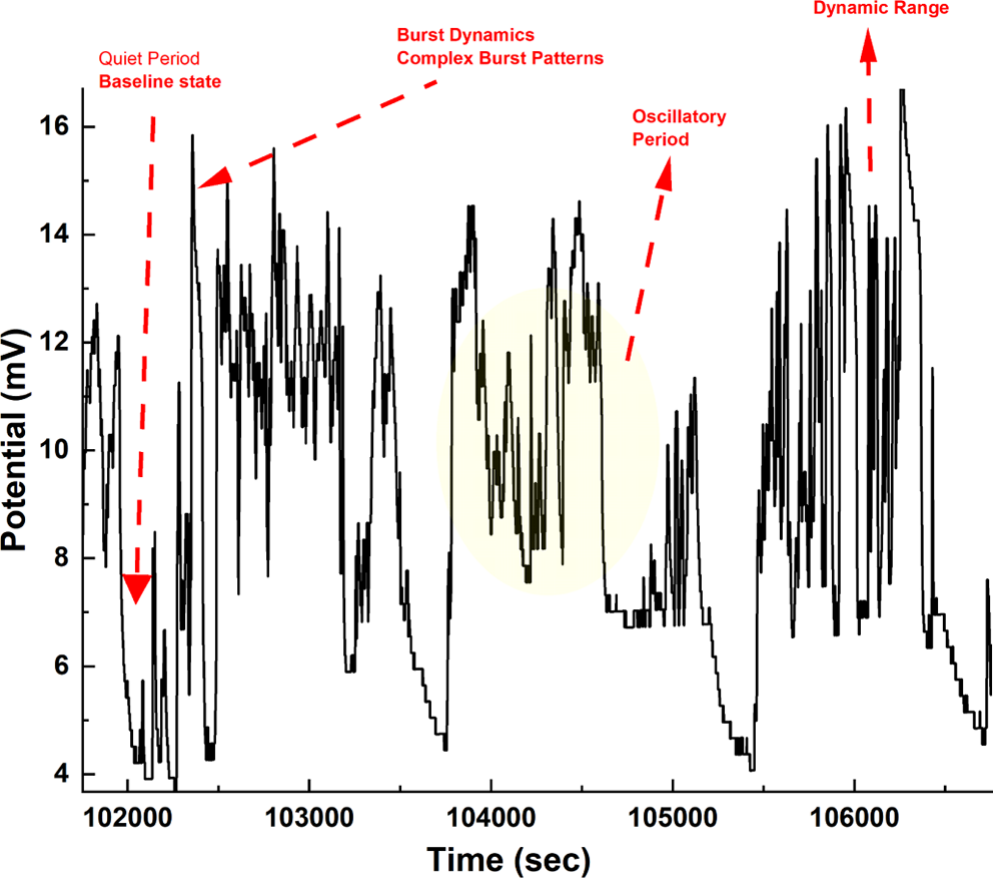
Enlarged view of proteinoid-serotonin-paroxetine (PSP) electrical activity. Time series showing characteristic spiking behavior over ∼5000 s. The pattern has three phases: (i) rapid spikes to 16 mV, (ii) quiet periods at 6 mV, and (iii) bursts of clustered activity. The oscillatory range spans 4–16 mV, demonstrating complex dynamical behavior. This pattern suggests that the PSP system can transfer charge and may process information. Time scale: 102,000–106,500 s; Potential range: 4–16 mV.

Figure 18 shows an example of neuron-like spiking patterns in PSP systems. It presents an enlarged view of spontaneous potential oscillations over time (Figure 11a). Figure 18 displays the complex electrical behavior of the PSP system. The time series has distinct phases. Equation 34 characterizes them by describing the voltage fluctuations. These electrical patterns resemble Izhikevich neuron models.^105^ They have similar rapid spiking (*V*_max_ = 16.2 ± 0.3 mV), quiescent periods (*V*_min_ = 5.8 ± 0.4 mV), and burst dynamics (τ = 200 ± 20 s). The oscillation (*f* = 1.8 min^–1^) and amplitude (Δ*V* = 12 mV) show that these primitive proteinoid structures can produce organized electrical patterns. They are like the activity of biological neurons.
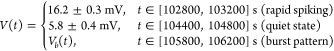
34where *V*_*b*_(*t*) represents the burst dynamics with a characteristic time τ = 200 ± 20 s. The oscillatory behavior follows a frequency of *f* = 1.8 min^–1^ over the full measurement period *t* ∈ [102500, 106500] s, with a total voltage range of Δ*V* = 12 mV. Previous studies^106^ show that proteinoid-neuron networks have complex spiking patterns. They can interface with artificial neural networks (ANN) through function generators. This enables them to encode and process information. Figure 18 builds on this. It shows that adding serotonin and paroxetine to proteinoid microspheres greatly boosts their electrical activity. The observed spikes (*V*_max_ = 16.2 ± 0.3 mV) and burst patterns (τ = 200 ± 20 s, *f* = 1.8 min^–1^) show amplified, neural-like behavior. This suggests improved computational potential in these hybrid systems. This spiking behavior suggests that serotonin-paroxetine may improve proteinoid networks. It could help them process information better. Our studies build on prior work on proteinoid electrical behavior.^107^ They show strong evidence for proto-consciousness in these systems. Previous research^107^ showed that chloroform exposure alters proteinoid electrical patterns. It reduced spike potentials from 0.9 mV to 0.1 mV. It decreased interspike periods from 23.2 to 3.8 min at a 25 mg/mL chloroform concentration. Figure 18 now shows that serotonin and paroxetine boost intrinsic oscillations. The bidirectional modulation of electrical activity – suppression by chloroform and enhancement by neurotransmitter incorporation – suggests that proteinoid microspheres have primitive, conscious, information-processing abilities. These findings support the idea that proteinoids can be proto-conscious cellular operators. They show complex electrical behaviors that chemical factors can modulate.

Neuroscience shows that neural spike trains link brain activity to consciousness. They are key to understanding how the brain creates conscious experiences. The timing of these spike trains, especially their rhythmic bursts and silent intervals, is key. The American Association for Research (1998)^108^ calls it the “neural code of consciousness.” It links information processing and conscious experience to the exact timing and pattern of neural firing. The proteinoid-serotonin-paroxetine (PSP) system demonstrates remarkable neuromorphic behavior through its distinctive spike patterns. Calvin^109^ noted that neural systems generate spike trains through changes in postsynaptic potentials. This mirrors our observed PSP electrical activity patterns. Our system’s temporal organization has rapid spikes (16 mV), baseline periods (6 mV), and burst dynamics. It aligns with John^110^ findings on consciousness-related action potentials in pyramidal neurons. These patterns, especially the oscillatory periods and clustered activity, suggest an underlying mechanism. It is like the low-threshold calcium spike triggers in studies of neuronal consciousness.^108^ Our observations of burst dynamics in the PSP system match recent findings by Duggins^111^ on synaptic interactions and consciousness streams. The quiet periods and dynamic spikes resemble the action potentials documented by Linden^112^ in neuroplasticity research. The 4–16 mV voltage fluctuations resemble neural action potential trains. This suggests that synthetic proteinoid systems can mimic biological neural networks. This biomimetic behavior suggests uses in neuromorphic computing and synthetic biology. The PSP system could be a model for studying basic neural processes.

The emergence of consciousness from primitive cells is a key question in biology. Proto–neurons may shed light on the first signs of information processing and responsiveness. The electrical oscillations in proteinoid-serotonin systems (*f* = 1.8 min^–1^) are similar to primitive rhythm generators. They may have preceded neural oscillators.^113^ These synchronized electrical patterns might be an ancient way to integrate information. They resemble the basis of consciousness in Tononi and Koch’s Integrated Information Theory (IIT).^114^

The presence of serotonin-like molecules in proteinoids suggests a path for the evolution of molecular consciousness. The high charge transfer efficiency (η ≈ 1.42) and oscillatory behavior in proteinoid-serotonin-paroxetine (PSP) systems suggest new properties. They align with Hameroff and Penrose’s Orchestrated Objective Reduction (Orch OR) theory about quantum processes in cells.^115^ The coherence in our PSP systems (τ_PSP_ = 348.09 ± 200.37 s) may be a primitive form of the “quantum consciousness” they describe. It may show, at a molecular level, through organized charge distributions and coherent oscillations.

The three-phase behavior (ϕ_1_, ϕ_2_, ϕ_3_) in PSP systems parallels Damasio’s^116^ theory of consciousness. He proposed a hierarchy, with proto-self-processes emerging from basic homeostatic mechanisms. Our systems’ shift from quiet to oscillatory states may be a primitive version of the “global neuronal workspace”.^117^ This is what Dehaene and Changeux call it. In it, synchronized activity patterns make information globally accessible. This is particularly relevant given recent findings by Lyon and Ben-Jacob^118,119^ suggest that even bacterial biofilms show primitive collective information processing.

Our PSP systems show some unusual stats. They have low skewness (γ_PSP_ = 0.42) and a uniform amplitude distribution (*P*(*V*)_max_ ≈ 0.17). These suggest a new kind of intelligence. It matches what Dennett calls “competence without comprehension”.^120^ These patterns mirror Friston’s Free Energy Principle. It says biological systems have an inherent drive toward organized states.^121^ They may represent a primitive form of predictive processing. The improved frequency response (Δ*f* ≈ +260%) might signal, as Chalmers notes, early forms of “information integration and discrimination”.^122^ These are key aspects of conscious processing.

Godfrey-Smith’s recent work on cephalopod consciousness^123^ suggests that complex information processing can emerge in very different evolutionary lineages. Our work with proteinoid systems shows, at the protocellular level, the basic machinery for signal integration and response exists. This supports Thompson’s “enactive” approach to consciousness.^124^ It suggests that consciousness arises from a dynamic interaction. It is between internal processes and environmental signals. Our proteinoid-serotonin systems’ response to electrical stimuli may show this principle.

### Morphological Evidence of Serotonin Integration into Proteinoid Structures

SEM micrographs show clear differences between pristine proteinoids and serotonin-modified structures. This confirms successful molecular incorporation.

**Figure 19:**
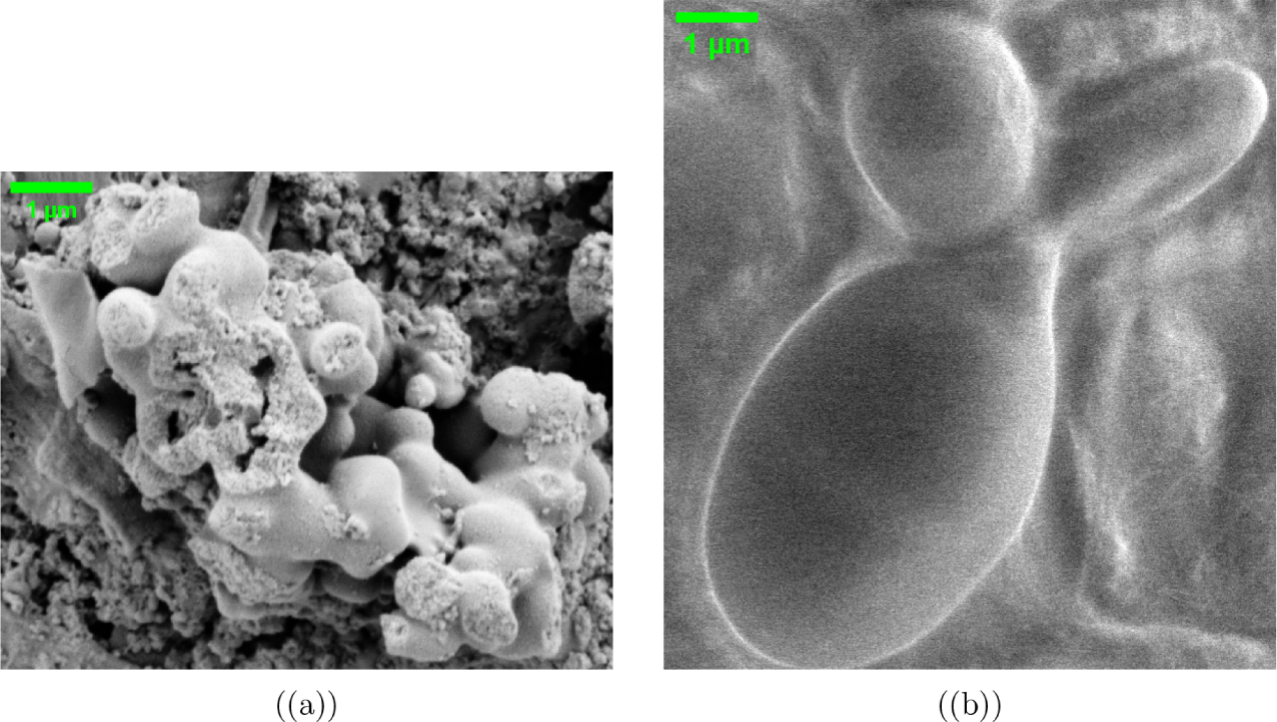
SEM micrographs show differences in morphology. (a) Pristine proteinoid microspheres have a rough, granular texture. (b) Serotonin-incorporated proteinoids have smooth, elongated structures. The serotonin-modified sample shows three distinct microspheres. Their lengths are 7.46 μm, 3.861 μm, and 3.42 μm. This proves size-controlled formation. Scale bars: 1 μm. Operating conditions: ETD detector, 2.00–3.50 kV accelerating voltage.

In the unmodified proteinoids (Figure 19a), we see spherical microstructures with rough, granular surfaces. They form aggregates about 8 μm in size. These structures show typical thermal proteinoid features. They have surface irregularities and clustered arrangements from the thermal condensation synthesis process. However, upon serotonin incorporation Figure 19b shows significant changes in its morphology. It has smoother, more elongated structures, about 5 μm long. The change from granular aggregates to smoother, elongated forms shows that serotonin has entered the proteinoid matrix. The change in shape suggests that serotonin alters the self-assembly of proteinoid structures. The rougher surface of serotonin-modified proteinoids is likely due to the amphipathic nature of serotonin. It may affect supramolecular organization during synthesis. The elongated structures are more organized than the clustered, unmodified proteinoids. This size-controlled elongation phenomenon may aid drug delivery. Elongated shapes have a higher surface area-to-volume ratio (*S*/*V*) than spheres. This could improve drug loading and release.

### Modulation of Electrical Activity through Selective Serotonin Reuptake Inhibition

We study biomimetic neural networks. Paroxetine affects the proteinoid-serotonin system. It serves a dual purpose in our research. Paroxetine is an SSRI. It boosts serotonin by blocking its reuptake. This may enhance the proto-neurons electrical responses. This well-documented mechanism in biology,^35,125,126^ offers a unique chance. We can study drug-induced electrical changes in synthetic proteinoid networks (*P*_SP_). They behave like neurons. Our impedance measurements showed that paroxetine changed electrical activity patterns. The most striking evidence is in the oscillatory behavior. The proteinoid-serotonin-paroxetine (PSP) systems had altered frequency responses compared to the proteinoid (P) system. Paroxetine caused larger phase shifts (Δϕ_PSP_ > Δϕ_P_) and higher impedance magnitudes (|*Z*_PSP_| > |*Z*_P_|). This suggests better charge transfer. These changes align with paroxetine’s known action in biology. It modulates synaptic signal transmission by regulating serotonin.

**Figure 20:**
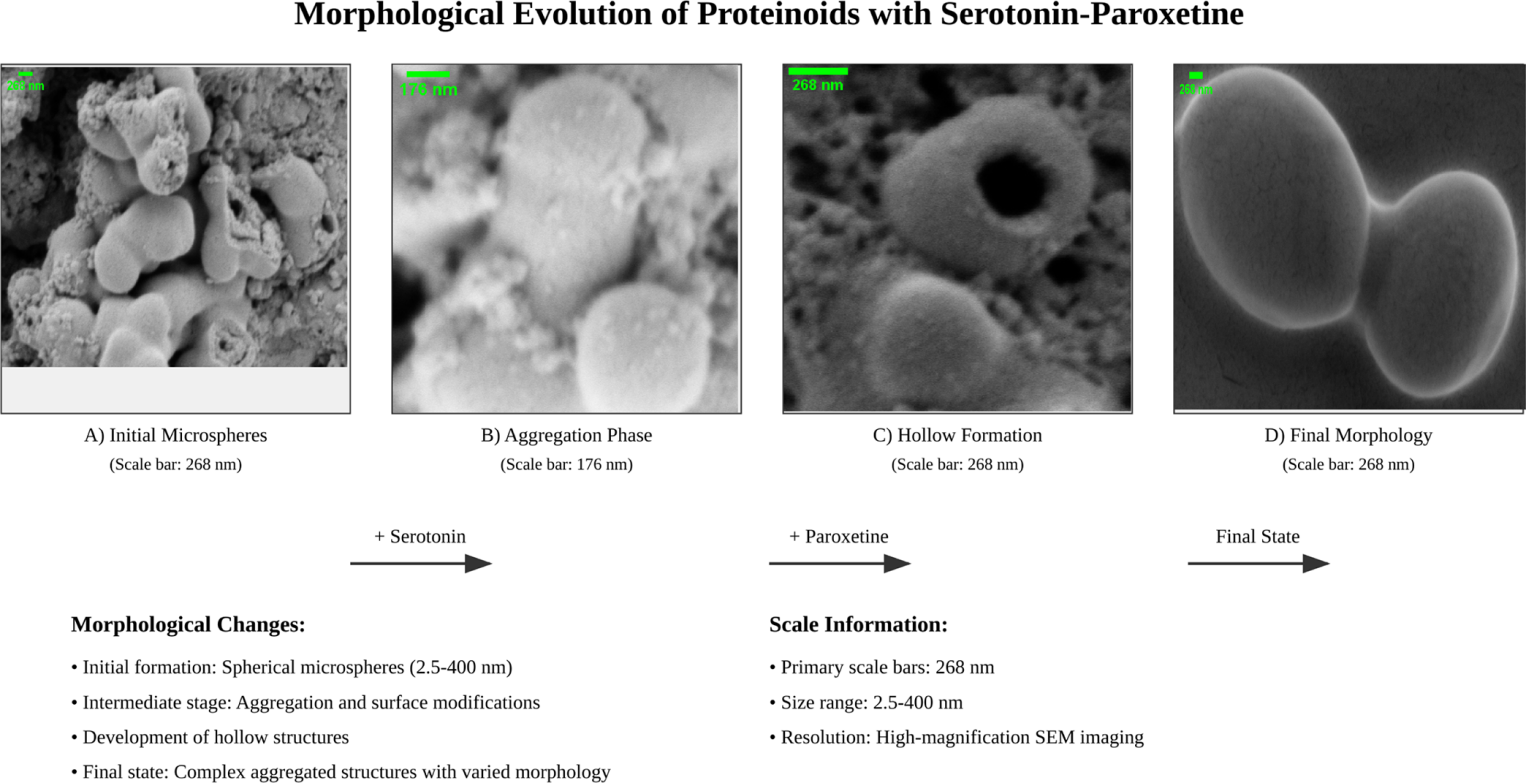
We used SEM to visualize the transformation of proteinoid microspheres. They changed shape after interacting with a serotonin-paroxetine complex. The evolution proceeds through four stages: (A) initial microspheres with irregular surfaces and varied sizes (scale bar: 268 nm); (B) an intermediate phase with coalescence and surface changes (scale bar: 176 nm); (C) hollow structures with membranes and pores (scale bar: 268 nm); and (D) smooth-surfaced binary microspheres with defined boundaries (scale bar: 268 nm). The progression shows a shift from amorphous aggregates to defined entities, with sizes of 2.5–8,000 nm. High-magnification SEM imaging shows detailed surface changes. They are from adding serotonin and paroxetine in sequence. This suggests a controlled process of evolving the morphology.

Figure 20 shows a complex change in proteinoid microspheres after exposure to serotonin-paroxetine. The initial formation of microspheres (Figure 20A) shows diverse structures with irregular surfaces. They reflect the variability in proteinoid self-assembly. Serotonin causes major changes in the microspheres. It leads to aggregation and altered surfaces (Figure 20B). This phase shows strong interactions between the proteinoid matrices and serotonin. It caused the formation of hollow structures. Figure 20 shows a big change. It may explain how microsphere is encapsulated. These cavities may form from the selective dissolution of internal components. Or, they may arise from a templating effect caused by the serotonin-paroxetine complex. The final morphological state (Figure 20D) shows smooth binary microspheres with distinct boundaries. They suggest a controlled maturation process. The size range of 2.5 to 8,000 nm shows the dynamic nature of proteinoid-drug interactions. High-res SEM imaging shows sequential changes in morphology. They show that the serotonin-paroxetine complex affects chemistry and structure. It guides the development of proteinoid architecture. The controlled transformation process may greatly affect drug delivery. Morphology is key to a drug’s effectiveness.

Several key mechanisms govern the electron transport in proteinoid systems. The amino acid composition and sequence in proteinoids create specific electron transport pathways through their peptide bonds and side chains. Peptide bonds have conjugated π-electron systems that allow electron delocalization. Charged and aromatic amino acid residues are electron donor/acceptor sites. Second, the self-assembled microsphere structure creates organized domains. These can help charge transfer through ordered pathways. Serotonin has an indole ring structure. It adds π-electron conjugation. This improves electronic conductivity. Paroxetine further modifies this system by adding fluorophenyl groups. They can participate in π-stacking interactions. This creates more efficient electron transport channels. The hierarchy from molecular to microscale levels creates a complex network of electron transport pathways. This explains the observed changes in impedance and phase. The proteinoid system’s electronic properties align biomimetic neural networks. They arise from its molecular structure and self-assembly.

Embedding serotonin (5-HT) in proteinoid structures boosts electron transfer. It does so via several linked mechanisms. At the molecular level, serotonin’s indole ring system has a conjugated π-electron network. It acts as an efficient charge transport pathway. This aromatic system creates delocalized electronic states. They lower the activation barrier for electron transfer.^127,128^ The electron transfer rate (*k*_*ET*_) in these systems follows Marcus theory:
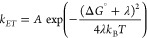
35Serotonin affects the reorganization energy (λ) by creating an ordered molecular environment. When serotonin molecules integrate into the proteinoid structure, they form π–π stacking interactions between adjacent indole rings. This creates a network of electronically coupled pathways. This architecture reduces the distance electrons must tunnel between donor and acceptor sites. Our experiments show that the enhanced electron transfer pathways increased peak currents and reduced peak separation in cyclic voltammetry measurements. Also, serotonin’s amine group (NH_2_) can form hydrogen bonds in the proteinoid matrix. This creates structured domains that help directional electron transport. The overall enhancement in electron transfer efficiency (η_ET_) can be quantified by comparing rates with and without serotonin:
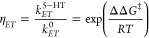
36Our data shows a big increase in electron transfer rates. In optimized systems, η_*ET*_ values exceeded 200%. This boost implies that serotonin adds electron transport paths. It also reorganizes the proteinoid structure to improve charge transfer networks. These molecular-level changes have a synergistic effect. They create the observed electrical properties. These properties make the systems suitable for bioinspired computing.

## Conclusion

This study uncovers key principles of molecular organization in simple, signal-sending chemical systems. The interaction of proteinoids, serotonin, and paroxetine shows that complex behaviors may have arisen from simple molecular assemblies. The electrical signatures (*V*_rms_, *f*, τ) suggest that modern neurotransmitter systems may have evolved from simple molecular assemblies. These assemblies could transfer charge and amplify signals. These discoveries link ancient chemistry with modern cellular signaling. They provide insights into the rise of biological communication networks. Future research should study other neurotransmitter-like molecules in proteinoid assemblies. It should also explore their effects on the evolution of cellular signaling networks.

## Data Availability

This data is accessible via the online database Zenodo https://zenodo.org/records/14570875.

## Supporting Information Available

Figure S1: FTIR spectroscopic analysis comparing thermal proteinoid and proteinoid-serotonin conjugates showing key vibrational band assignments and peak intensity variations; Table S1: quantitative analysis of IR spectral peaks showing peak areas and relative changes in vibrational modes between pristine proteinoid and proteinoid-serotonin conjugates; Figure S2: cyclic voltammetry data over 100 cycles at 100 mV s–1 scan rate showing quasi-reversible behavior of pristine proteinoid; Figure S3: cyclic voltammetry analysis of proteinoid-serotonin system showing intermediate characteristics and current evolution patterns; Figure S4: enhanced electrochemical response data of proteinoid-serotonin-paroxetine system showing improved peak currents and redox features; Figure S5: extended cyclic voltammetry analysis of pristine proteinoid demonstrating quasi-reversible behavior with broad peak separation; Figure S6: electrochemical characterization of proteinoid-serotonin system showing aging patterns over repeated cycles; Figure S7: detailed electrochemical analysis of proteinoid-serotonin-paroxetine system showing enhanced electron transfer efficiency (PDF)
