# Supplementary material for: Serotonergic Mechanisms in Proteinoid-Based Protocells
Source: ACS Chem Neurosci. 2025 Jan 22;16(3):519–42. doi: 10.1021/acschemneuro.4c00801 (PMC11803625; doi:10.1021/acschemneuro.4c00801)
Supplement: Supplementary file 1 — cn4c00801_si_001.pdf [file cn4c00801_si_001.pdf]

Supporting Information

Serotonergic Mechanisms in Proteinoid-Based Proto-Cells

Panagiotis Mougkogiannis[1,*] and Andrew Adamatzky[1]

[1]Unconventional Computing Laboratory, University of the West of England, Bristol, UK, BS16 1QY

Email: Panagiotis.Mougkogiannis@uwe.ac.uk

1 FT-IR Spectroscopy

The FTIR analysis (Figure S1) shows that we successfully made proteinoid structures and modified them with serotonin. The thermal proteinoid spectrum shows the peptide backbone absorption bands. The strong Amide I band at 1650 cm^{-1} indicates mainly α-helix and random coil forms. Serotonin's incorporation into the proteinoid structure is evident. There are notable spectral changes, especially in the fingerprint region (1500-1000 cm^{-1}). The increased absorption and slight band shifts in the Amide I and II regions of the proteinoid-serotonin conjugate suggest successful coupling. They may also indicate a change in the proteinoid's structure from the serotonin.

The two spectra differ in peak intensities and shapes, especially in the 1800-1000 cm^{-1} region. This further validates the chemical modification of the proteinoid backbone. The preserved amide bands in both spectra confirm that the peptide structure is intact after serotonin conjugation. The spectral differences show that serotonin moieties were successfully incorporated into the proteinoid framework. These spectral features support a stable proteinoid-serotonin conjugate. They also maintain the essential proteinoid structure.

Table S1 shows the analysis of key vibrational bands. It shows large increases in peak areas across all modes. The amide regions show a dramatic enhancement that stands out. The Amide I and Amide III bands increased by 617.4% and 658.8%, respectively. The strong rise in the amide bands shows a major change in the peptide backbone's shape. The N-H stretching intensity rose by 380.6%. This suggests a strong hydrogen bonding network. The CH_2 bending mode increased by 555.2%. This shows big changes in aliphatic chain interactions. These spectral changes provide strong evidence that serotonin was integrated. They also indicate a complete reorganization of the proteinoid architecture. This likely improved molecular interactions and stability.

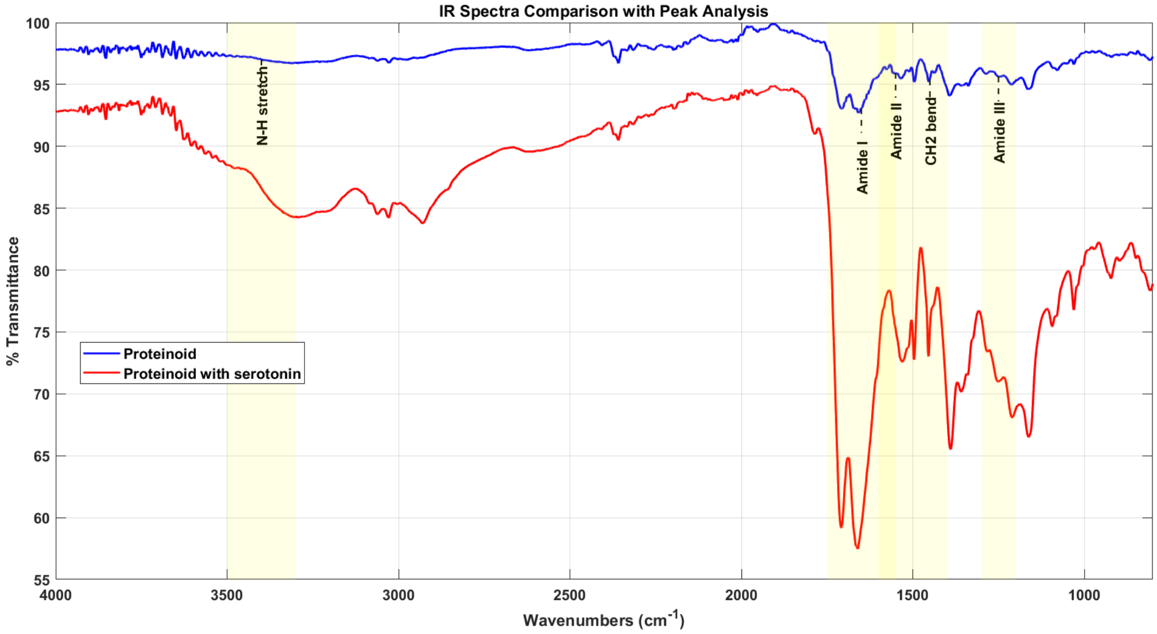

Figure S1: FTIR spectra comparing thermal proteinoid (blue line) and proteinoid-serotonin conjugate (red line). The spectra show peptide bonds with prominent amide bands: Amide I (1650 cm^{-1}, C=O stretching), Amide II (1550 cm^{-1}, N-H bending and C-N stretching), and Amide III (1250 cm^{-1}, C-N stretching and N-H bending). The N-H stretching vibration at 3400 cm^{-1} and CH_2 bending at 1450 cm^{-1} further confirm the proteinoid structure.

Table S1: Quantitative analysis of IR spectral peaks comparing pristine proteinoid and proteinoid-serotonin conjugates. We calculated peak areas from absorbance spectra. We used trapezoidal integration over the vibrational bands. The peak areas show big increases after adding serotonin. This means there were major structural changes and better molecular interactions. This is especially true in the peptide backbone regions (Amide I, II, and III) and N-H stretching modes. The spectral integration shows that all characteristic peaks rose significantly after adding serotonin. The most pronounced changes are in the Amide I (617.4%) and Amide III (658.8%) regions. They indicate major changes in the peptide backbone's conformation. The N-H stretch's intensity rose by 380.6%. This suggests extensive hydrogen bonding. The CH_2 bending mode increased by 555.2%. This reflects changed aliphatic chain interactions. These spectral changes show successful serotonin integration. They also show a big restructuring of the proteinoid architecture.

**Vibrational Mode**	**Position (cm^{-1})**	**Peak Area**		**Relative Change (%)**
		**Proteinoid**	**With Serotonin**	
N-H stretch	3400	2.61	12.57	+380.6
Amide I	1650	4.64	33.27	+617.4
Amide II	1550	1.74	12.23	+602.6
CH_2 bend	1450	1.76	11.51	+555.2
Amide III	1250	1.91	14.46	+658.8

2 Cyclic Voltammetry

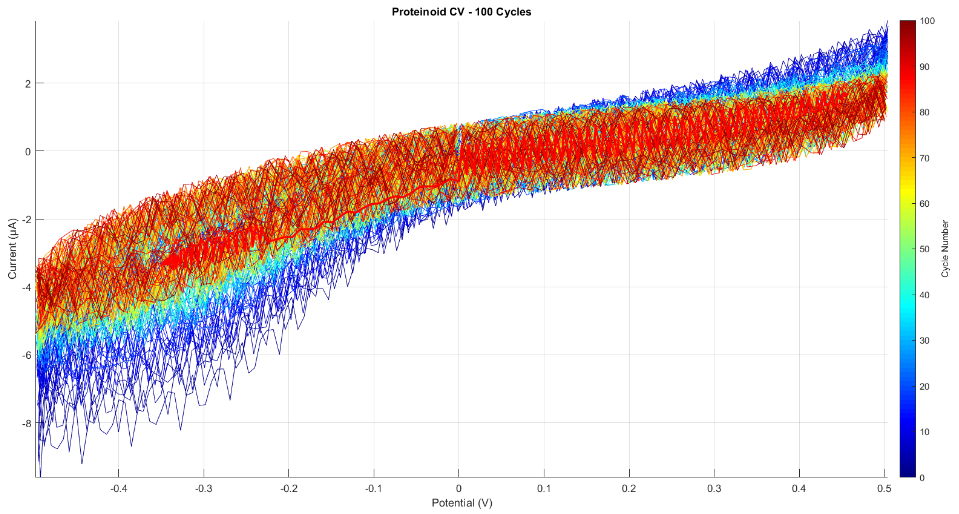

Figure S2: Comparative cyclic voltammetry analysis of proteinoid systems over 100 cycles at 100 mV s^{-1} scan rate. Pristine proteinoid showing quasi-reversible behaviour with peak currents ranging from -8 to +3 μA and $\Delta E_p = 0.958 \pm 0.033$ V. The broad potential window (-0.5 to +0.5 V) exhibits significant current dispersion across cycles, indicating complex electron transfer dynamics.

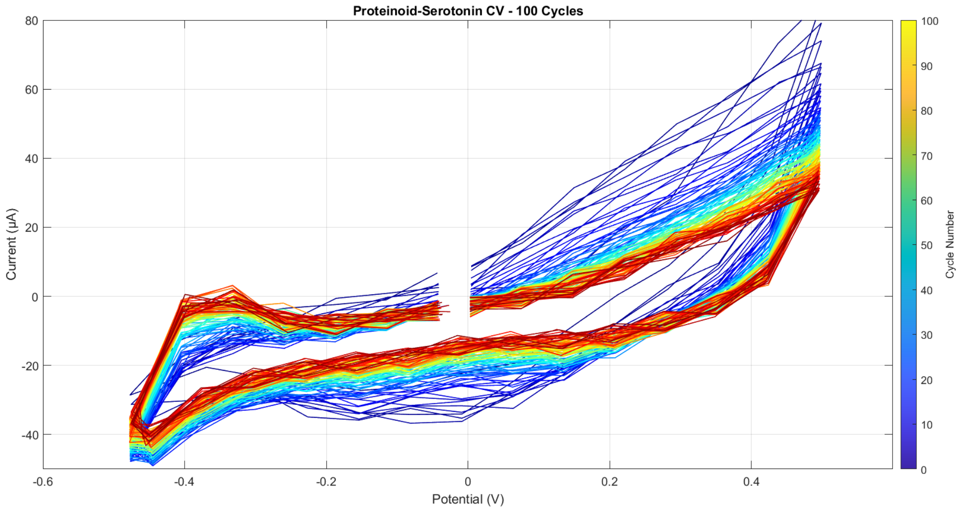

Figure S3: Proteinoid-serotonin system displaying intermediate characteristics with current ranges from -20 to +80 μA. The colour gradient represents cycle progression (blue$\rightarrow$red: cycles 1$\rightarrow$100), revealing intermediate organization in current evolution.

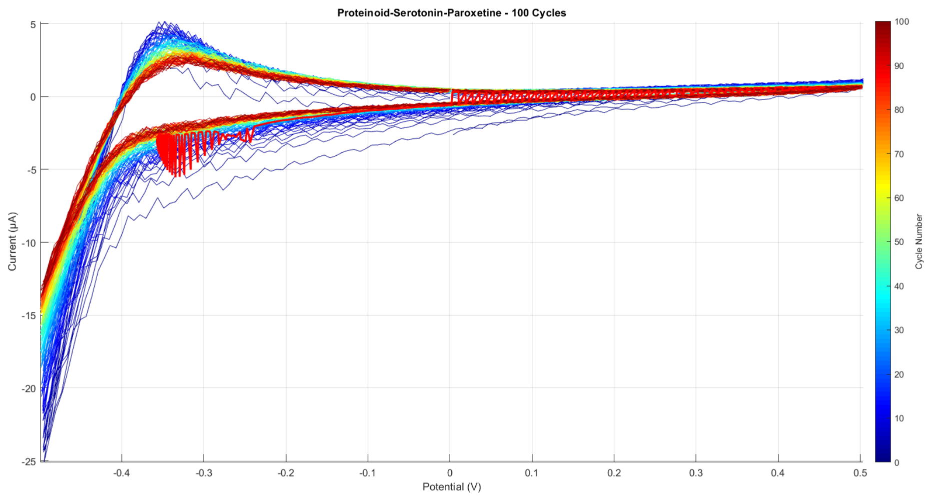

Figure S4: Proteinoid-serotonin-paroxetine system demonstrating enhanced electrochemical response with higher peak currents (-25 to +5 μA) and sharper redox features ($\Delta E_p = 0.166 \pm 0.013$ V). The colour gradient represents cycle progression (blue→red: cycles 1→100), revealing organized current decay, suggesting structured electron transfer pathways. The progressive improvement in reversibility and current magnitude through the series indicates that both serotonin and paroxetine incorporation fundamentally alter the electron transfer mechanism, creating more efficient charge transport channels in the proteinoid matrix.

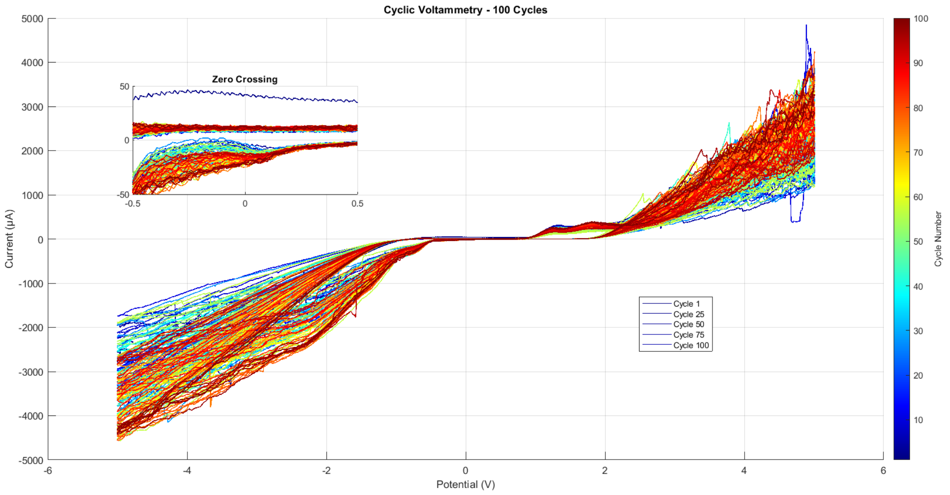

Figure S5: Cyclic voltammetry analysis of pristine proteinoid over 100 cycles showing quasi-reversible behavior with broad peak separation ($\Delta E_p = 0.958 \pm 0.033$ V) and current extremes ranging from -4500 to +4500 μA. The inset shows zero-crossing region ($\pm$0.5 V), revealing moderate hysteresis. Scan rate: 100 mV s^{-1}, potential window: $\pm$5 V vs. Ag/AgCl.

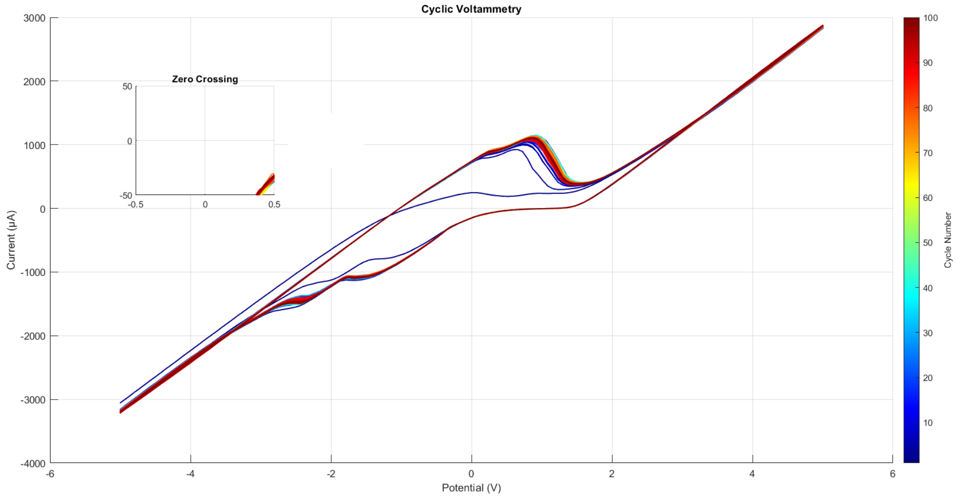

Figure S6: Proteinoid-Serotonin system demonstrating intermediate electrochemical characteristics with current range (-3200 to +2900 μA). Colour gradients represent cycle progression (blue$\rightarrow$red: cycles 1$\rightarrow$100), revealing distinct aging patterns. Scan rate: 100 mV s^{-1}, potential window: $\pm$5 V vs. Ag/AgCl.

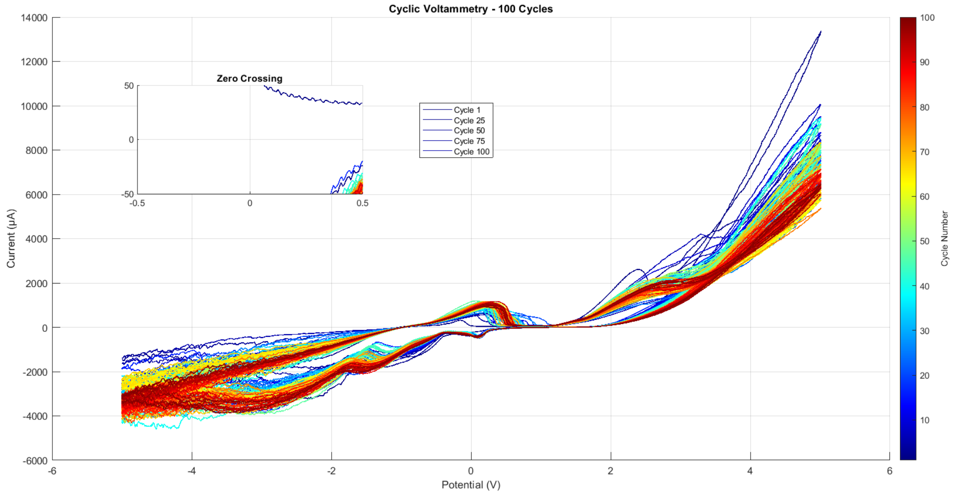

Figure S7: Proteinoid-Serotonin-Paroxetine system exhibiting enhanced electrochemical response with reduced peak separation ($\Delta E_p = 0.166 \pm 0.013$ V) and increased current range (-6000 to +13000 μA). Colour gradients represent cycle progression (blue$\rightarrow$red: cycles 1$\rightarrow$100). The system demonstrates $\sim$2200% enhancement in electron transfer efficiency ($\epsilon_{\text{prot-sero}}/\epsilon_{\text{prot}} = 23.03$), suggesting formation of ordered charge transport pathways through serotonin-mediated molecular organization. Scan rate: 100 mV s^{-1}, potential window: $\pm$5 V vs. Ag/AgCl.
